# A quantitative classification of OTC medicines regulations in 30 European countries: dispensing restrictions, distribution, pharmacy ownership, and pricing systems

**DOI:** 10.1186/s40545-023-00522-7

**Published:** 2023-01-30

**Authors:** Eduardo Daniel López Vila, Caroline Buts, Marc Jegers

**Affiliations:** Department of Applied Economics, Vrije Universiteit Brussel, Pleinlaan 2, 1050 Brussels, Belgium

**Keywords:** OTC medicines, Europe, Cluster analysis, Regulatory framework

## Abstract

**Background:**

This paper reviews the regulations of over-the-counter (OTC) medicines in 30 European countries with the goal of identifying the regulatory trends and clusters as of May 2022.

**Methods:**

To that end, we reviewed the regulation that directly or indirectly might have an impact on OTC medicines. The data were gathered from the national legislation, reports from international organizations, and the existent literature. The 12 regulatory items obtained were classified into four categories: price, pharmacy ownership, distribution modes, and dispensing restrictions. In addition, these items were also employed in the cluster analysis.

**Results:**

Pharmacy ownership is mainly private, and in the majority of countries, OTC medicines are not subject to any pricing system. Almost every country studied allows online selling of OTC medicines, and 16 countries allow non-pharmacy retail to sell OTC medicines as well. The dispensing restrictions applicable in pharmacy retail are similar in the countries studied: they rely on the staff, OTC medicines are placed behind the counter and the doses dispensed tend to be restricted. Concerning non-pharmacy retail, additional dispensing restrictions might be imposed, such as the establishment of buyers’ minimum age, the requirement of a pharmacist to supervise the operations, a regulation on the location in the store, and further restrictions on the package sizes, strength, or pharmaceutical form. The cluster analysis resulted in an initial division between countries that widely allow the sale of OTC medicines in non-pharmacy retail and countries, where pharmacy retail has an OTC monopoly. Based on the regulations, 7 subsequent groups were identified evidencing wide regulatory heterogeneity within the countries studied.

**Conclusions:**

Our findings point out that OTC medicines are in general not subject to pricing systems, selling is allowed online, and ownership of pharmacies is mostly private. However, regarding dispensing restrictions, pharmacy chains, and establishment restrictions of pharmacies, we found heterogeneity that is also visible in our cluster analysis, since we identified 7 clusters.

**Supplementary Information:**

The online version contains supplementary material available at 10.1186/s40545-023-00522-7.

## Background

In general, European countries are moving toward the liberalisation of several aspects of the regulatory framework of over-the-counter (OTC) medicines, such as pricing and distribution modes. A clear example is the lifting of the pricing system of OTCs in Greece in mid-2017 [1], or the recent deregulation of the distribution modes in Lithuania in 2019, where the Government allowed non-pharmacy retail to sell OTC medicines. Exceptionally, there are a few countries like Poland and Hungary that took the opposite direction, from deregulated systems to a more regulated system, where pharmacies are only owned by pharmacists and retail pharmacy chains are not allowed or limited [2].

To our knowledge, a review of the regulatory framework for OTC medicines in the European region is not yet available and some topics such as dispensing restrictions in non-pharmacy retail remain even unexplored. However, the literature widely reviews the regulatory framework of community pharmacies, and casually, several authors refer to OTC medicines. The report from the World Health Organization (WHO) [3] reviews the regulatory framework of community pharmacies in 2018 for 49 countries. It reviews the requirements to own a community pharmacy, establishment criteria, and other regulations, such as horizontal and vertical integration restrictions. It updates and enlarges Martins et al. [4] who reviewed the regulatory framework in 2013 of the European community pharmacies in 19 countries. Member States of the European Union (EU) should also follow certain additional regulations and directives issued by the European institutions to harmonise the internal market. Some relevant examples are the European Directive 2001/83/EC in which the criteria for classifying a medicinal product as prescribed or OTC was introduced. The latter was amended by the European Directive 2011/62/EU in which a code to prevent falsified products into the supply chain was proposed, and the common logo was first introduced with the goal to identify the websites that are legally selling medicinal products through online channels. Similarly, the European Regulation 699/2014 further defines and designs the common logo to identify the person/entity selling medicinal products.

The regulatory framework of the distribution channels of OTC medicines in European countries was reviewed by Vogler [5] and Oleszkiewicz et al. [6], both papers classifying the European countries into three groups. The first group includes countries, where the distribution is monopolised by community pharmacies. Second, the sale of OTC medicines is allowed in community pharmacies and non-pharmacy retailers (e.g., supermarkets, gas stations, etc.). Third and last, the sale of OTC medicines is limited to non-pharmacy retailers. Differences in distribution channels and the regulatory frameworks of community pharmacies in Europe and their impact on prices, and accessibility are studied by several authors [7–10]. The impact on prices is mixed, and accessibility increases mostly in urban areas, while in rural areas remains unchanged or decreases. The authors also point out that a wrong design of the ownership may lead to an oligopoly and thus erode competition. Anell [11] introduces the cases of Iceland and Norway. Both countries deregulated the ownership and competition rules to foster competition, but instead, ended up with an oligopoly, where two or three pharmacy groups owned 85% and 97% of the market, respectively.

Finally, the studies that cover the prices of OTC medicines are rather limited. Vogler [12] states that the scarcity of such studies might be due to the fact that prices of OTC medicines are generally not regulated, and price data are not available or too expensive to be purchased. An exception is Stargardt et al. [13] who studied the deregulation of the prices of several OTC substances in Germany in 2004 finding that 2 years after the deregulation only a few pharmacies used individual pricing strategies. In other words, price competition was not the rule. Consequently, the literature indicates that deregulation does not necessarily lead to lower pharmaceutical expenditure, lower prices, or higher accessibility. Additional topics such as analysis of switching medicines from prescribed to OTC, the implementation of dispensing restrictions, and its impact on the safety of the patients are also reviewed by several authors [14–17]. The fact that the literature only covers specific topics related to OTC medicines shows the need for a comprehensive review of the regulatory frameworks and their classification.

Therefore, this paper aims, first, to review the current regulatory framework around OTC medicines including pricing systems, distribution modes (pharmacy and non-pharmacy retail, whether the selling of OTC medicines is allowed online or not), ownership and establishment of pharmacy retail, and the additional dispensing restrictions that must be adopted in the non-pharmacy retail, such as age restriction, dosage restriction (size of the packs, pharmaceutical forms, strength, etc.). Second, performing a classification of the 30 countries studied (the Member States of the European Union, Norway, Switzerland, and the United Kingdom) based on the review of the regulatory framework which is intended to help future researchers to assess the impact of such regulations on prices, accessibility, along with other indicators or dimensions.

The remainder of the paper is organized as follows. Section “Data and methodology” introduces and describes the data collection and the methodology employed for the clustering process. The third section is divided into two subsections. The first describes the results obtained from the review of several regulatory acts and described the regulatory trends within the 30 countries studied. The second subsection introduces the results obtained from the clustering process and the dimensions of such classification. The fourth section provides a discussion of the results and the last section underlines the main conclusions.

## Data and methodology

This section introduces the data gathering process, as well as the clustering methodology employed. The review of primary and secondary sources to perform the review was also used in Leopold et al. [18] Oleszkiewicz et al. [6], or reports from international entities, such as the WHO or the OECD. The data gathering process is divided into the following steps:

1. Review the current regulation that might directly and/or indirectly impact OTC medicines:
  - Regulation retrieved at the national level of the countries studied from legal documents and different ministries, such as the Ministry of Finance, Ministry of Health, and other national authorities.
  - Reports from international organizations, such as the WHO, the Organisation for Economic Co-operation and Development (OECD), the Pharmaceutical Group of the European Union (PGEU), the European Commission (EC), and the European Medicines Agency (EMA) among others.
  - Existent literature that reviews several aspects of the regulatory framework that might have an impact on OTC medicines (e.g., [1, 2, 19, 20]), mainly retrieved from PubMed and Scopus.
2. Produce a summary for each country.
3. Identify the main regulatory trends and items in each of the countries.
4. Extraction of the regulatory items.
5. Distribution of the items into categories.

The same process is applied to the regulation of each of the countries, ensuring a systematic review process that allows comparability between countries. Whenever there was a conflict between national legislation and other resources, national legislation was given primacy over the other resources. The items retrieved are transformed into a binary variable that takes 0 if there is no regulation on this item and 1 if it is regulated (e.g., if the country has no pricing systems established for OTC medicines then the value would be 0) until we end up with a database with only binary variables. Several decisions were taken while building the database such as simplifying some of the regulatory items. Whenever a regulatory item is simplified additional analysis and explanations will be given in the third and fourth sections of the paper. We follow the recommendations obtained from a Monte Carlo simulation performed by Tamasauskas et al. [21] and thus employ the complete linkage as a clustering algorithm and Sokal and Sneath 1 as the distance measurement.

## Results

We divide the result section first introducing the results of the review of the regulatory framework of OTC medicines, and second, the results of the cluster analysis based on the regulatory review.

### OTC regulatory framework review

The review of the regulation was performed between January and mid-May 2022. More than 200 documents were reviewed (including international reports, existent literature, national legislation, etc.). The approach followed was described in the second section and exactly the same process was followed for each of the countries. In Additional file 1: Appendix 1 we present the sources reviewed and a table that describes the number of resources and the type of resources reviewed for each country. Some of them were also used during the paper, and therefore, these sources were also included in references. While reviewing the documents, we identified that the regulatory items found could be classified into the following four categories: dispensing restrictions, distribution modes, ownership, and pricing systems. A total of twelve regulatory items were found and distributed into these four categories, these are presented in Table 1.

**Table 1:** Categories and regulatory items

Category	Regulatory items
Price	Pricing of OTC medicines is free or subject to a pricing system
Ownership (Pharmacy retail)	Public or private ownership
	Private ownership limited to only pharmacists or open to third parties
	Pharmacy chains allowed or not
Distribution	Online distribution allowed or not
	OTC medicines are available only at pharmacy retail or if non-pharmacy retail is also allowed to sell OTC medicines
	Pharmacy retail has establishment restrictions (Distance, inhabitants) or freedom of establishment
Dispensing restrictions (Non-pharmacy retail)	Supervision of the pharmacists is required
	Accessibility and/or location are regulated
	Minimum legal age
	Only drugstores or specialised stores
	Dosage restrictions (e.g., size of the pack, strength, or pharmaceutical form)

As to regulations related to dispensing OTC medicines in pharmacy and non-pharmacy retail, pharmacy retail is subject to more or less the same regulation in the countries studied, as it relies on the staff of the pharmacy and the pharmacists that supervise the operations of the pharmacy, as well as the fact that the accessibility to OTC medicines is in general restricted to the public. When non-pharmacy retail is allowed we find several regulations designed to ensure the safeness and health of the consumer when acquiring OTC medicines that might be applied in these shops: the imposition of the minimum legal age to acquire OTC medicines, supervision of the operations of the shop by a pharmacist to ensure its proper functioning and dispense the OTC (these regulations are not present in all the countries). In 13 of these countries, the dosage of OTC medicine is restricted (in comparison with the pharmacy retail) in terms of quantities, strengths, and forms that present a very limited risk for the person. In addition, accessibility and storage are regulated in some countries to have OTC medicines located behind the counter, on shelves with lockers, or another restriction that ensures proper dispensing of the medicine. We find that all countries that opened the distribution of OTC medicines to non-pharmacy retail are applying at least one of these regulations.

Three of the ten items identified were classified in the category distribution modes. The three items were whether OTC medicines could be sold online or not, whether the selling of OTC medicines was allowed in the non-pharmacy retail, and last, if the establishment of pharmacy retail was subject to restrictions, such as the number of inhabitants, distance, etc. The restriction or lack of restriction of the establishment of pharmacies does have an impact on the accessibility of OTC medicines in countries, where non-pharmacy retail is not allowed, an example of their impact of them can be found in Barbarisi et al. [26]. Regarding the online selling of OTC medicines, we find a clear picture of their availability online in 28 of the 30 countries, which differs from the prescribed medicines, where online selling is allowed in less than 15 countries and with several restrictions. Greece and Cyprus are the only countries that are not harmonised with the European Directive 2011/62/EU and the European Regulation 699/2014 regarding the common logo that should be displayed in online pharmacies since the selling of OTC medicines and in general medicinal products through online channels are not allowed. Therefore, their so-called online pharmacies are not displaying the logo on their websites. Although Cyprus attempted to harmonize its regulation setting the necessary conditions for the sale of medical products online, the reality is that according to the Ministry of Health of Cyprus the national legislation states that medicinal products can only be dispensed personally by the pharmacist making acquiring medical products through online channels illegal.

Concerning the physical distribution modes, we find that 16 countries allow non-pharmacy retail to sell OTC medicines. In the remainder of the countries studied, the distribution of OTC medicines is a monopoly or almost a monopoly of the pharmacies. With respect to the establishment rules for pharmacies, the results obtained are also similar to the ones obtained in previous studies [3, 5]. In 13 of the countries studied there are no establishment rules for pharmacies while in 17 at least they have a need assessment or, distance and/or inhabitant rule that must be followed to open a new pharmacy. A summary of the distance and population restrictions as well as needs assessments to establish a new pharmacy is presented in Additional file 1: Appendix 2.

The ownership of pharmacies is mainly private in 26 of the countries studied. Only in Denmark, Luxembourg, Slovenia, and Finland countries, where the ownership of pharmacy retail is limited to the state, in other words, the owner is the state and the state is the one that issues concessions of the pharmacies to pharmacists [3, 27]. These four countries are the only ones, where the pharmacies are exclusively public, while in other countries such as Italy and Sweden public ownership is allowed [9, 28]. In addition, in the countries, where the ownership of pharmacies is private, we have to distinguish between countries where the ownership is limited to only pharmacists and countries where the ownership of a pharmacy is open to non-pharmacist parties. To point out that in the majority of the countries there are rules established to avoid vertical (e.g., pharmaceutical companies), and horizontal (e.g., limited number of pharmacies or maximum market share) integration to avoid scenarios presented in previous studies (e.g., Norway, or Iceland) [3, 11, 28]. Of the 27 countries that allow private ownership 9 limit it to only pharmacists and 18 do not. It is necessary to point out that although these 18 countries do not limit the ownership it is clearly stated that a pharmacist should supervise the operations of the pharmacy or own some of the shares (e.g., In Greece a pharmacist should hold at least 20% of the shares [29]).

In addition, we include in the category ownership the possibility of establishing pharmacy chains. We find that pharmacy chains are allowed in 17 countries. The other 13 countries are restricted. The criteria employed to determine that in a country pharmacy chains were not allowed if the regulation was not explicitly mentioned it was the fact that the pharmacy ownership was limited. We consider that the country was implicitly defining a pharmacy chain as the maximum number of pharmacies that can be owned by the same person/entity plus 1. To clarify, if the regulation states that the same person/entity can own up to 4 pharmacies, then implicitly the country defines a pharmacy chain as 5 or more pharmacies. In Additional file 1: Appendix 3 a summary of the regulations related to pharmacy chains is presented. Ownership was considered relevant for the analysis as the literature evidences that in countries like Poland or the US, independent pharmacies are overall achieving higher patient satisfaction, as well as performing better in other areas, such as counselling [30, 31]. In addition, the staff of pharmacy chains deal with higher levels of stress as was found in England and the US [32, 33], which may lead to lower performance, lower job satisfaction, and thus lowering the patient satisfaction. In addition, some issues related to the autonomy of the pharmacist might arise while working in large pharmacy chains. Finally, in countries where the ownership is public or restricted to pharmacists pharmacy chains were forbidden.

The prices of OTC medicines are not regulated in 24 of the 30 countries analysed. Belgium, Bulgaria, Latvia, Lithuania, Luxembourg, and Finland, are the only countries that still regulate the prices of OTC medicines. A maximum ex-factory price is set by the Belgian national authorities [22], and the margins for wholesalers and pharmacists have been set as a function of the ex-factory price, allowing price competition if desired. In Finland, the national authorities set the ex-factory prices of OTC medicines, and these prices must be the same for all pharmacies and online [23]. In Bulgaria, the maximum retail prices are subject to registration by the National Council on Prices and Reimbursement of Medicinal Products (NCPRMP), and no other external price reference system is used as explained in Dimova et al. [24]. Finally, the retail maximum prices of OTC medicines in Latvia are set by a process that involves the manufacturer, and the marketing authorization holders (MAH), details about the pricing process can be found in Silins and Szkultecka-Dębek [25].

The results obtained from the review of the regulatory framework identified the existent trends in the 30 countries studied. The majority of the countries do not fix the price of OTC medicines and allow selling through online channels. Half of the countries liberalised the physical distribution of OTC medicines to non-pharmacy retail imposing restrictions to limit the risk associated with OTC medicines. The most common ones are restrictions on package size, strength, and pharmaceutical form. Finally, the ownership of pharmacies is mainly private and pharmacy chains are allowed in 17 of the countries studied.

### Classification based on the regulatory framework

**Fig. 1:**
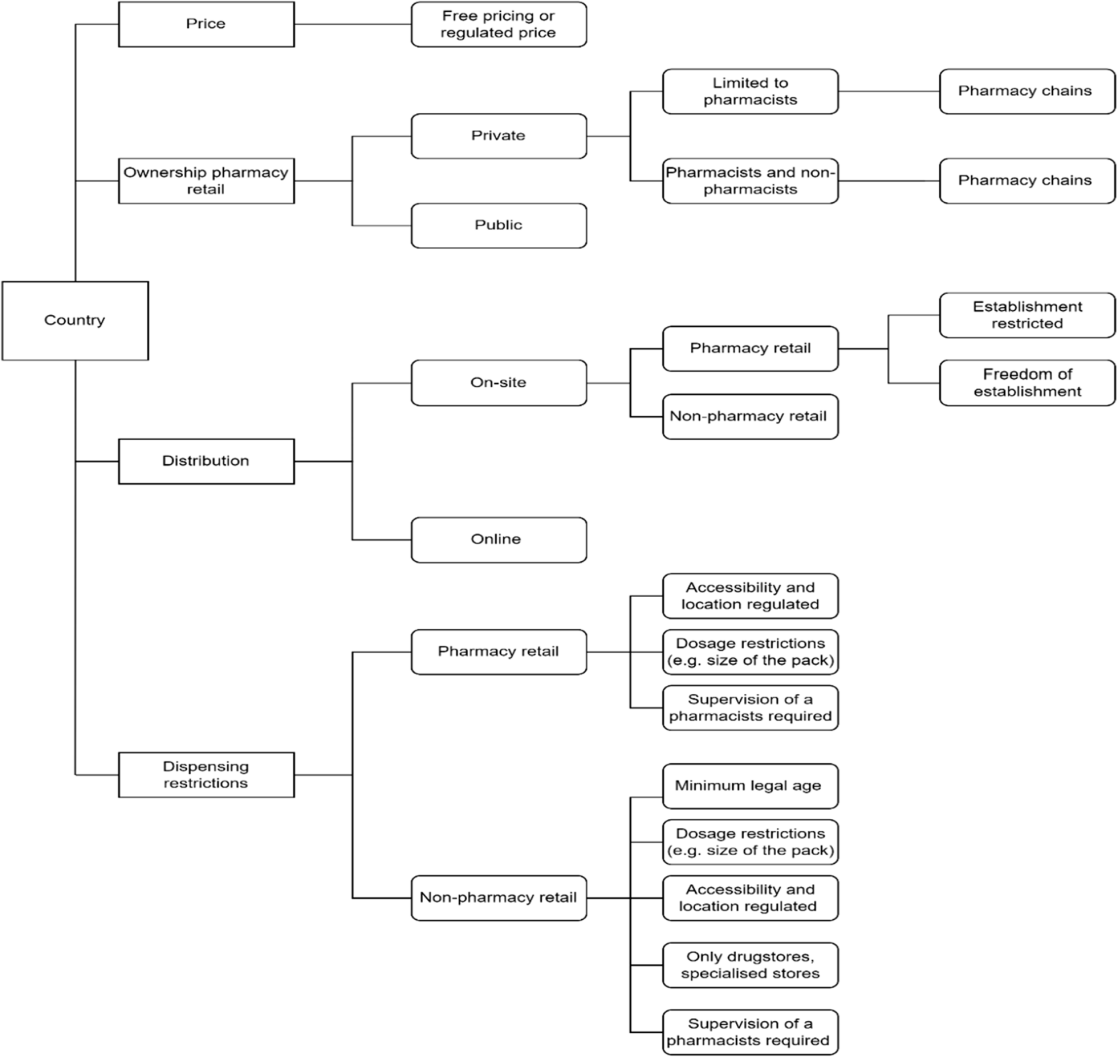
Regulatory framework

The review of the regulatory framework of these 30 European countries allows us to study whether we can identify clusters of countries that apply similar regulations using hierarchical analysis. Figure 1 introduces an overview of the regulatory framework and the potential regulations that the different countries may adopt and the different regulatory items within the categories. Additional examples are presented in Additional file 1: Appendix 4, Fig. 1 is tailored based on the regulation of each country.

We consider it necessary to split the countries studied into two groups. In the first group, pharmacy retail has the monopoly or almost the monopoly of the OTC market, since the range of products that can be sold outside pharmacies is rather limited. The countries that have allowed the selling of OTC medicines also in non-pharmacy retail were classified in the second group. It is logical and necessary to make such a division, as the regulation applied in non-pharmacy retail is not applicable in pharmacy retail, therefore, in countries, where non-pharmacy retail is not allowed such regulations are not present. In countries like Bulgaria, you can find OTC medicines in non-pharmacy retail but it is so limited that it results in almost a monopoly of pharmacy retail [5]. Another example is Germany, as according to section 50 of their medicinal products law you can find OTC products in non-pharmacy retail but it is limited to disinfectants intended for external use, oxygen, and products to prevent pregnancy and sexually transmitted diseases. Therefore, countries in which the trade and the range of products outside pharmacies are so limited were included in the first group. The dendrograms on which the cluster classification and maps are based are presented in Additional file 1: Appendix 5.

**Fig. 2:**
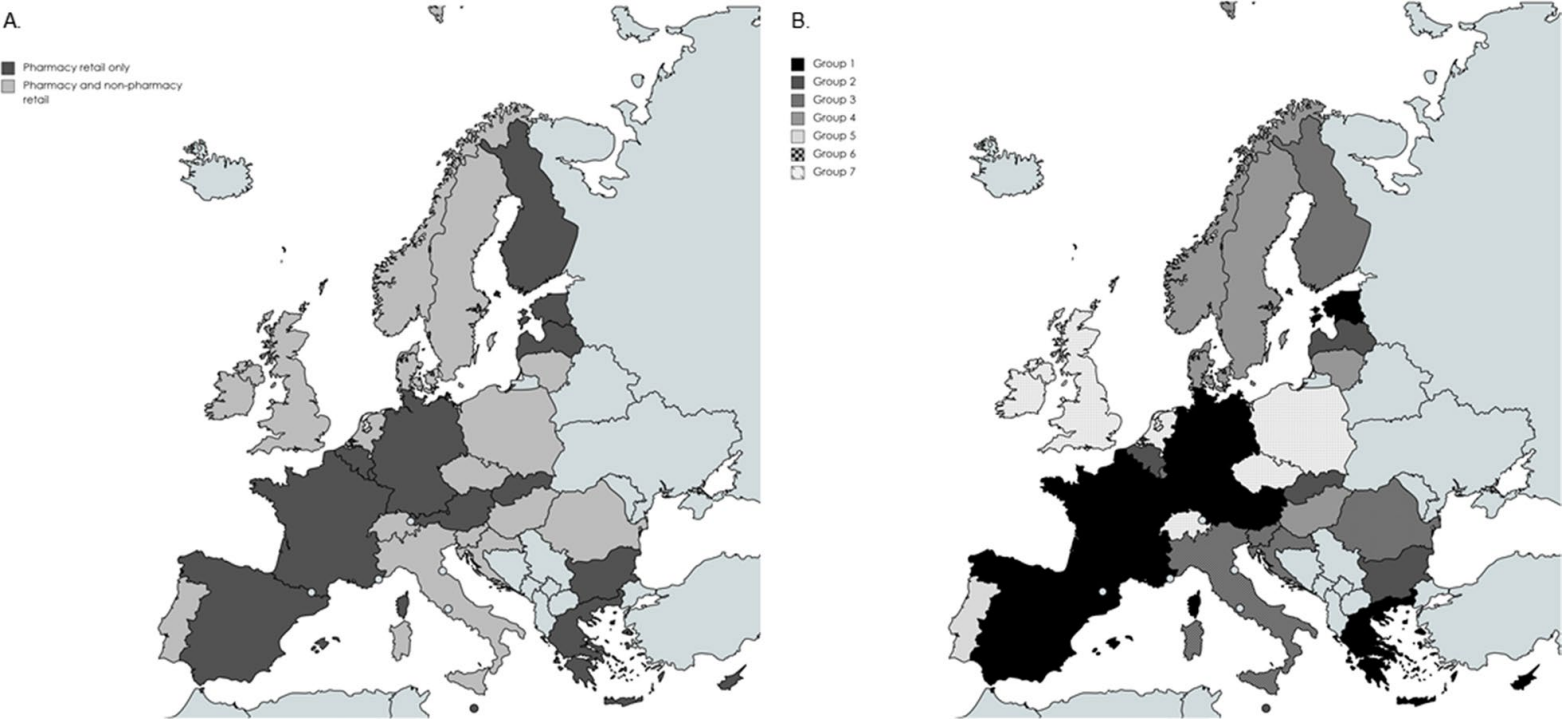
Maps of country groups

Figure 2A shows the geographical distribution of the countries after dividing them into countries, where pharmacy retail has the monopoly and almost the monopoly of the market and countries, where non-pharmacy retail was fully allowed. It is visible that western, north-eastern, and south-eastern Europe countries do not allow non-pharmacy retail, while in the majority of Nordic countries, this is allowed. In addition, Fig. 2B presents the number of groups obtained after applying hierarchical analysis in each of the two groups, resulting in 8 subgroups.

#### Pharmacy retail only

**Fig. 3:**
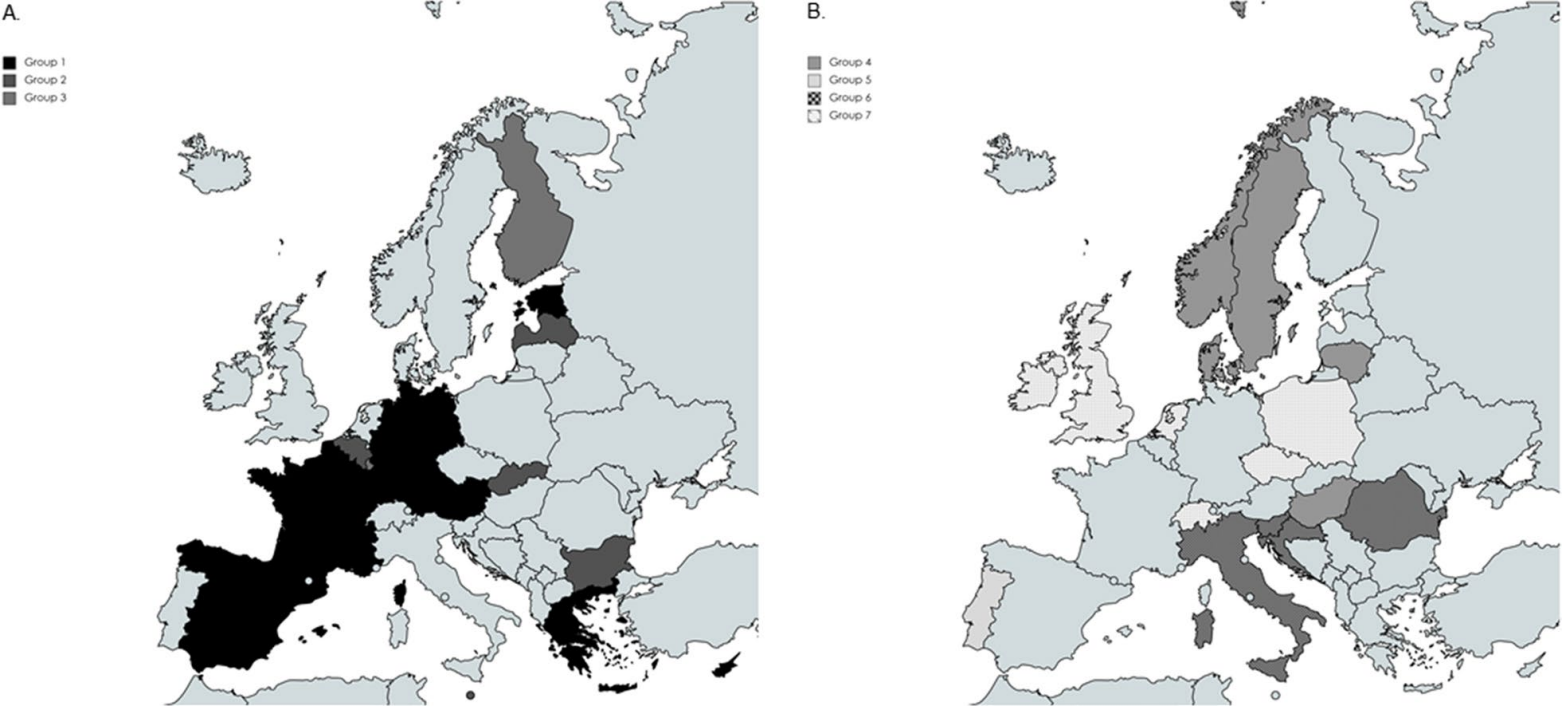
Maps of subgroups

The dendrogram presented in Additional file 1: Appendix 5 evidence a division between countries that allow pharmacy chains and countries that do not. The final classification show 3 groups. The results obtained are presented in Fig. 3A. The first group consists of 7 countries in which the prices of OTC medicines are not fixed, pharmacy chains are not allowed, and OTC can be bought through online channels in 5 of the 7 members (Greece and Cyprus do not allow online selling). The establishment of community pharmacies is restricted in 4 countries, two of these countries applied establishment restrictions based on distance and inhabitants requirements and the other two restricts the establishment based on inhabitants only. Pharmacies are owned privately in all of them (6 of them limit the ownership to pharmacists, and the other one allows non-pharmacists to own a pharmacy as long as a pharmacist has at least 20% of the shares).

The second group is formed by 5 countries. All of them permit the acquisition of OTC medicines through online channels. The ownership is private in all of them, the ownership of a pharmacy is open to pharmacists and non-pharmacists, and pharmacy chains are allowed. The majority of the members restrict the establishment of pharmacies based on inhabitants and distance requirements. In three of the countries prices of OTC medicines are regulated.

The third group is made up of two countries. Both of them have a mechanism that fixes the prices of OTC medicines, the ownership of the pharmacies is public, the state is exclusively the owner of the pharmacy, and the one that issues the concession that pharmacists can acquire to run a pharmacy. In addition, both allow the selling of OTC medicines through online channels, and both impose restrictions on the establishment of pharmacies. In Finland, they impose restrictions based on the number of people and distance of health services, while Luxembourg bases the issue of new pharmacy concessions based on demographics.

#### Pharmacy and non-pharmacy retail allowed

To perform the cluster analysis, we include variables related to dispensing restrictions in non-pharmacy retail. The variables classified in the category of dispensing restrictions are determinants of the groups, which also differ from the previous groups, and can be due to the fact that these countries apply a rather similar regulation in the other categories. In total, 4 groups were identified, from which 3 are groups of 4 or more countries and one consists of an outlier country (Portugal). Groups are visible in Fig. 3B.

The first group of this category or the fourth group of the analysis comprises 5 countries. The establishment of pharmacies is free in the majority of countries, and the ownership of the pharmacies is private in 4 of them. In Denmark, the owner of the license is the state, and the state lets a pharmacist operate the license until the pharmacist retires, dies, or acquires a new pharmacy. Again, pharmacy chains are allowed in countries, where the ownership is not limited to pharmacists or the state (in other words, pharmacy chains are allowed in 3 countries). With respect to dispensing restrictions to non-pharmacy retail, in all of them, minimum legal age applies and restrictions on the size of the packages and the strength of the product are imposed, as well as restrictions on the accessibility of OTC medicines to limit the risk, which might be due to the fact that the operations of the non-pharmacy retail in these countries do not need to be supervised by a pharmacist.

The fifth group consists of one country, Portugal. It does not allow pharmacy chains, although the ownership of the pharmacies is not limited to pharmacists. In addition, it imposes several dispensing restrictions on the non-pharmacy retail such as establishing a minimum age to acquire OTC medicines, and supervision by a pharmacist is required to ensure the adequate functioning of the section, where OTC medicines are placed as the dosage is not restricted and the accessibility is not regulated.

The sixth group is made up of 4 countries that restrict the establishment of pharmacies, and the ownership of pharmacies is mostly private. The countries do not impose a minimum age to acquire OTC medicines in non-pharmacy retail, but the operations of the non-pharmacy retail must be supervised by a pharmacist or qualified personnel, and accessibility is restricted. In 3 of the 4 countries, the availability of OTC medicines is limited to only drugstores that are supervised by qualified personnel.

In the seventh group, the six members allow pharmacy chains, pharmacies are owned by private parties, and the majority do not impose any restrictions on the establishment of new pharmacies. With regard to the dispensing restrictions, members of this group do not impose as many restrictions as the other ones. Neither minimum age nor supervision of the pharmacist is required, and there are no regulations imposing accessibility restrictions. The only dispensing restriction in all of the members are restrictions related to strength, package size, and pharmaceutical forms of OTC medicines allowed in the non-pharmacy retail.

## Discussion

The review of the regulatory framework of OTC medicines in 30 European countries showed a clear trend of liberal pricing systems as the majority of the countries do not impose any kind of mechanism to set prices. Examples of countries with liberalised pricing systems are: Italy since 1995, Germany since 2004, Portugal since 2005, and Greece in mid-2017. Even though, the Greek authorities publish an indicative retail price. There are currently six countries that still regulate prices: Belgium, Bulgaria, Finland, Latvia, Lithuania, and Luxembourg. Deregulating the price is one of the most common measures adopted to reduce pharmaceutical expenditure, lower prices, and foster price competition. However, according to Stargardt et al. [13], the deregulation of the price of OTC medicines did not lead to lower prices 2 years after the liberalisation in Germany, suggesting that competition was not in prices but rather in services.

Furthermore, the number of European countries allowing non-pharmacy retail to sell OTC medicines is increasing. Maybe in a near future, more countries will debate the liberalisation of the distribution modes. The latest country that opened the distribution of OTC medicines to non-pharmacy retailers was Lithuania in 2019, increasing the number of European countries that allow non-pharmacy retail to widely sell OTC medicines to 16 countries. The liberalisation of the distribution of OTC medicines through non-pharmacy retailers is quite specific in each country, as there are countries that fully liberalised the distribution of certain OTC medicines, such as the Netherlands, Norway, or the UK, while other countries have “liberalised” the distribution in a limited way like in Bulgaria, where you can sell a limited number of medicines through vending machines that should be owned by a pharmacist making the trade of OTC medicines outside pharmacies residual or almost non-existent. The liberalisation of the distribution of OTC medicines is usually driven by policymakers stating that such a policy will bring a reduction in prices, as well as increase accessibility and competition. However, the existent literature provides mixed results. Vogler et al. [7] and Vogler et al. [9] state that prices do not show a significant decrease after the deregulation of distribution the accessibility only increases in urban areas. Lluch and Kanavos [8] found that countries such as Spain with the regulated establishment of pharmacies based on demographics or geographic criteria lead to higher equity of access. In addition, the authors found that in the UK as supermarkets tend to offer cheaper OTC medicines than pharmacies, resulting in significant savings [8]. On the contrary, Moura and Barros [10] found that in Lisbon supermarkets on average offer 20% lower prices than pharmacies and foster price competition, since pharmacies close to supermarkets decrease their prices only between 4% and 6%. However, according to the authors, this is not the general rule but is dependent on the local situation.

Concerning the ownership of pharmacies and the existence of pharmacy chains in the country, we identify some discrepancies and movements in both directions. Although in the majority of countries, the ownership of pharmacies is open to non-pharmacists and pharmacy chains are allowed, there are some movements toward the restriction of the ownership to only pharmacists and a restriction of the activities of pharmacy chains. A clear example is Hungary, which gradually shifted from a liberalised system, where pharmacy chains were allowed and the ownership was open to third parties to a system, where pharmacy chains were outlawed and pharmacists should own at least 51% of the pharmacy by 2017. A similar situation is observed in Poland with the introduction in 2017 of an amendment to the Pharmaceutical Law according to which the ownership of a pharmacy can only be sold to a pharmacist or group of pharmacists. Concerning pharmacy chains, the amendment allows existing pharmacy chains to operate but restricts their expansion [2].

The regulatory trends are also visible from the cluster analysis we performed, resulting in 7 regulatory subgroups. The cluster analysis of the countries, where pharmacy retail is a monopoly showed that the regulatory items within the categories of price, ownership, and distribution play a crucial role in the classification of the countries. The latter is not the case in the 16 countries, where non-pharmacy retail is allowed, as 15 of them do not regulate the price, all of them allow selling OTC medicines through online channels, and ownership in almost all of them is private, in other words, these countries do have similar regulations in three of the four categories identified. Therefore, dispensing restrictions play a vital role in the determination of the composition of the groups.

At last, the heterogeneity that is obtained from the review and the subsequent cluster analysis should point out that higher convergence in terms of regulation should be achieved between the countries of the European region, but also the EU. We acknowledge that such convergence should be achieved within the two main groups, countries, where pharmacies have the monopoly of dispensing OTC medicines, and countries, where non-pharmacy retail is widely spread and is allowed to dispense OTC medicines. In other words, in countries, where pharmacy retail has the monopoly, the European institutions along with national authorities should harmonise the policies applied regarding pricing systems, the establishment of pharmacy retail, as well as ownership with the aim of increasing drug safety, accessibility, and ensuring the proper functioning of the market. Similarly, in countries, where non-pharmacy retail is allowed to sell OTC medicines, there should be higher convergence regarding the dispensing restrictions applied. We consider that these 16 countries should converge toward a unique policy mix, in which safety is the main goal. For example, countries in the 7th cluster should move toward the policy mix applied in the 4th group if the supervision of qualified staff is not required through the establishment of a minimum legal age to purchase OTC medicines and regulating the accessibility of them, so that the intervention of the staff is required to purchase them. However, the best safety measure to be applied is to ensure that the presence of a pharmacist is required to sell OTC medicines outside pharmacy retail. At last, there is an important heterogeneity between the countries studied and it should be mentioned that further convergence in terms of regulation should be achieved at least between the countries that are part of the EU. In other words, countries, where OTC medicines area monopoly of the pharmacies, should move toward a common policy mix, and countries, where you can find OTC medicines outside pharmacies should provide a common policy mix that ensures the safety of the patient above everything as well as reducing the potential shock that the patients may face when moving from one country to another.

## Conclusion

To conclude, the regulatory items identified during the review of the regulation of the European countries studied were classified into four categories. In the majority of countries prices of OTC medicines are not determined by any national authority, the selling of OTC medicines is allowed through online channels, and pharmacy retail is mainly owned by private actors. Private ownership could be limited to only pharmacists or open to other private actors, and pharmacy chains are not allowed in countries, where the ownership is public or limited to pharmacists. In addition, we find that 16 of the 30 countries allow the selling of OTC medicines in non-pharmacy outlets. The countries that restrict the establishment of pharmacy retail employ restrictions based on needs assessments, inhabitants, or/and distance. The countries that allow non-pharmacy retail to sell OTC medicines are most likely to impose additional dispensing restrictions on non-pharmacy retail, such as accessibility restrictions, the establishment of minimum legal age, or restricting the size of the package, the strength, or the pharmaceutical form of the OTC medicine in the non-pharmacy retail. In addition, the cluster analysis evidences the existence of 7 subgroups within the two main groups. The first 3 subgroups are determined based on the restrictions imposed on prices, ownership, or distribution modes. In contrast, in the other 4 subgroups, clusters were mainly driven by dispensing restrictions as the majority of the countries apply similar regulations in terms of ownership, and distribution.

We acknowledge some limitations: the cluster methodology is sensitive to outliers, the transformation of the regulatory items into dichotomous variables reduces, simplifies the information, and ignores some details of the regulation that may be due to the particularities of the country. The result of the present paper is a snapshot of the current regulation, which in the future might change. The results obtained open new research avenues to study the impact of the regulatory framework on prices, accessibility, safety, as well as on the density of pharmacists and pharmacies based on the classification obtained or with regard to a specific policy mix, regulatory item, or category.

### Limitations

We acknowledge some limitations: the cluster methodology is sensitive to outliers, the transformation of the regulatory items into dichotomous variables reduces the information, and might ignore some details of the regulation, potentially due to the particularities of the country. Finally, the present paper is a snapshot of the current regulation, which in the future might change.

## Supplementary Information

**Additional file 1. Appendix 1**. Sources employed in the review of the regulatory framework. **Appendix 2**. Establishment restrictions for pharmacy retail. **Appendix 3**. Summary of pharmacy chains regulation. **Appendix 4**. Examples of the application of the regulatory framework. **Appendix 5**. Dendrograms.

## Data Availability

The data set used and analysed for this study is available upon request.
