# Supplementary material for: A quantitative classification of OTC medicines regulations in 30 European countries: dispensing restrictions, distribution, pharmacy ownership, and pricing systems
Source: J Pharm Policy Pract. 2023 Jan 30;16:19. doi: 10.1186/s40545-023-00522-7 (PMC9887745; doi:10.1186/s40545-023-00522-7)

Appendix 1: Sources employed in the review of the regulatory framework

Table A.1. Summary of the resources employed

| **Country** | **National Legislation** | **International Organisations** | **Existing Academic Literature** |  |
| --- | --- | --- | --- | --- |
|  |  |  |  |  |
| Austria | 5 | 1 | 5 |  |
| Belgium | 7 | 1 | 4 |  |
| Bulgaria | 5 | 1 | 5 |  |
| Croatia | 6 | 2 | 6 |  |
| Cyprus | 4 | 1 | 3 |  |
| Czech Republic | 5 | 1 | 4 |  |
| Denmark | 8 | 1 | 4 |  |
| Estonia | 5 | 1 | 4 |  |
| Finland | 6 | 2 | 2 |  |
| France | 9 | 1 | 3 |  |
| Germany | 6 | 2 | 3 |  |
| Greece | 8 | 2 | 3 |  |
| Hungary | 8 | 1 | 3 |  |
| Ireland | 6 | 1 | 5 |  |
| Italy | 10 | 1 | 5 |  |
| Latvia | 7 | 1 | 4 |  |
| Lithuania | 8 | 1 | 3 |  |
| Luxembourg | 8 | 1 | 3 |  |
| Malta | 4 | 1 | 2 |  |
| Netherlands | 6 | 1 | 4 |  |
| Norway | 9 | 1 | 3 |  |
| Poland | 8 | 1 | 4 |  |
| Portugal | 14 | 1 | 4 |  |
| Romania | 5 | 1 | 3 |  |
| Slovakia | 4 | 1 | 3 |  |
| Slovenia | 9 | 1 | 4 |  |
| Spain | 6 | 1 | 4 |  |
| Sweden | 7 | 1 | 5 |  |
| Switzerland | 6 | 2 | 4 |  |
| UK | 7 | 1 | 3 |  |

**Common**

ABDA. (2021). *Density of Pharmacies in the EU*. ABDA. Retrieved May 4, 2022, from <https://www.abda.de/en/pharmacies-in-europe/density-of-pharmacies-in-the-eu>

Martins, S. F., Van Mil, J. W., & Da Costa, F. A. (2015). The organizational framework of community pharmacies in Europe. *International journal of clinical pharmacy*, *37*(5), 896-905. <https://doi.org/10.1007/s11096-015-0140-1>

Morthorst, B. R., Erlangsen, A., Nordentoft, M., Hawton, K., Hoegberg, L. C. G., & Dalhoff, K. P. (2018). Availability of paracetamol sold over the counter in Europe: a descriptive cross‐sectional international survey of pack size restriction. *Basic & Clinical Pharmacology & Toxicology*, *122*(6), 643-649. <https://doi.org/10.1111/bcpt.12959>

OECD & European Union. (2020). *Health at a Glance: Europe 2020: State of Health in the EU Cycle*. OECD Publishing, Paris. <https://doi.org/10.1787/82129230-en>

OECD. (2021). *Health at a Glance 2021: OECD Indicators*. OECD Publishing, Paris. <https://doi.org/10.1787/ae3016b9-en>

Oleszkiewicz, P., Krysinski, J., Religioni, U., & Merks, P. (2021, February). Access to Medicines via Non-Pharmacy Outlets in European Countries—A Review of Regulations and the Influence on the Self-Medication Phenomenon. In *Healthcare* (Vol. 9, No. 2, p. 123). Multidisciplinary Digital Publishing Institute. <https://doi.org/10.3390/healthcare9020123>

Vogler, S. (2014). Liberalization in the pharmacy sector. OECD Global Forum on Distribution.

World Health Organization. Regional Office for Europe. (‎2019)‎. *The legal and regulatory framework for community pharmacies in the WHO European Region*. World Health Organization. Regional Office for Europe. <https://apps.who.int/iris/handle/10665/326394>

**Austria**

Langer, T., Spreitzer, H., Ditfurth, T., Stemer, G., & Atkinson, J. (2018). Pharmacy practice and education in Austria. *Pharmacy*, *6*(3), 55. <https://doi.org/10.3390/pharmacy6030055>

BWB (2018). Sector Inquiry Health. Part I: The Austrian Pharmacy Market, BWB/AW-431, Vienna.

*Bundesrecht konsolidiert: Gesamte Rechtsvorschrift für Apothekengesetz*. (2021). Rechtsinformationssystem. Retrieved March 31, 2022, from <https://www.ris.bka.gv.at/GeltendeFassung.wxe?Abfrage=Bundesnormen&Gesetzesnummer=10010169>

*Bundesrecht konsolidiert*. (2015, June 25). Rechtsinformationssystem. Retrieved March 31, 2022, from <https://www.ris.bka.gv.at/eli/bgbl/1983/185/P59/NOR40170422?Abfrage=Bundesnormen&Kundmachungsorgan=&Index=&Titel=Arzneimittelgesetz&Gesetzesnummer=&VonArtikel=&BisArtikel=&VonParagraf=59&BisParagraf=&VonAnlage=&BisAnlage=&Typ=&Kundmachungsnummer=&Unterzeichnungsdatum=&FassungVom=23.03.2022&VonInkrafttretedatum=&BisInkrafttretedatum=&VonAusserkrafttretedatum=&BisAusserkrafttretedatum=&NormabschnittnummerKombination=Und&ImRisSeitVonDatum=&ImRisSeitBisDatum=&ImRisSeit=Undefined&ResultPageSize=100&Suchworte=&Position=1&SkipToDocumentPage=true&ResultFunctionToken=9f119e31-31f4-470e-bef0-f307077ad5ce>

Austria-Forum. (2020, January 22). *Medikamentenabgabe*. Retrieved March 31, 2022, from <https://austria-forum.org/af/AustriaWiki/Medikamentenabgabe>

*Bundesrecht konsolidiert: Gesamte Rechtsvorschrift für Arzneimittelgesetz*. (2022). Rechtsinformationssystem. Retrieved March 31, 2022, from <https://www.ris.bka.gv.at/GeltendeFassung.wxe?Abfrage=Bundesnormen&Gesetzesnummer=10010441>

*Austrian medicinal product index – Online Search for medicinal products*. (n.d.). Austrian Medicines and Medical Devices Agency. Retrieved March 31, 2022, from <https://aspregister.basg.gv.at/aspregister/faces/aspregister.jspx>

**Belgium**

SPF Economie. (2018, July 10). *Médicaments en vente libre ou médicaments OTC (Over The Counter) | SPF Economie*. Retrieved January 23, 2022, from <https://economie.fgov.be/fr/themes/ventes/politique-des-prix/prix-reglementes/medicaments-usage-humain/medicaments-originaux/medicaments-en-vente-libre-ou>

Agence Fédérale des Médicaments et des Produits de Santé (AFMPS). (n.d.). *Réglementation - Médicaments par internet*. AFMPS. Retrieved January 23, 2022, from <https://www.campagnesafmps.be/fr/medicaments-par-internet/reglementation>

Agence Fédérale des Médicaments et des Produits de Santé (AFMPS). (n.d.-a). *Pharmacies ouvertes au public*. AFMPS. Retrieved January 23, 2022, from <https://www.afmps.be/fr/humain/medicaments/medicaments/distribution/pharmacies_ouvertes_au_public>

Agence Fédérale des Médicaments et des Produits de Santé (AFMPS). (2022, January 20). *Arrêté royal concernant l’enregistrement et la répartition des officines ouvertes au public, et abrogeant les arrêtés royaux du 25 septembre 1974 concernant l’ouverture, le transfert et la fusion d’officines pharmaceutiques ouvertes au public et du 21 septembre 2004 relatif au transfert d’une officine ouverte au public vers un bâtiment d’un aéroport*. MONITEUR STAATSBLAD BELGE. Retrieved May 15, 2022, from <http://www.ejustice.just.fgov.be/cgi_loi/change_lg.pl?language=fr&la=F&table_name=loi&cn=2022011601>

Agence Fédérale des Médicaments et des Produits de Santé (AFMPS). (2021, April 7). *Lois et Arrêtés*. AFMPS. Retrieved January 23, 2022, from <https://www.afmps.be/fr/items-HOME/Lois_et_Arretes>

Agence Fédérale des Médicaments et des Produits de Santé (AFMPS). (n.d.-a). *Banque de données des médicaments*. AFMPS. Retrieved January 23, 2022, from <http://banquededonneesmedicaments.fagg-afmps.be/>

Centre Belge d’Information Pharmacothérapeutique (CBIP). (n.d.). *Répertoire*. Retrieved January 23, 2022, from <https://www.cbip.be/fr/chapters>

**Bulgaria**

Dimova, A., Rohova, M., Atanasova, E., Kawalec, P., & Czok, K. (2017). Drug policy in Bulgaria. *Value in Health Regional Issues*, *13*, 50-54. <https://doi.org/10.1016/j.vhri.2017.08.001>

Petkova, V., & Atkinson, J. (2017). Pharmacy practice and education in Bulgaria. *Pharmacy*, *5*(3), 35.  <https://doi.org/10.3390/pharmacy5030035>

министъра на здравеопазването. (2008, December 9). *НАРЕДБА № 28 ОТ 9 ДЕКЕМВРИ 2008 Г. ЗА УСТРОЙСТВОТО, РЕДА И ОРГАНИЗАЦИЯТА НА РАБОТАТА НА АПТЕКИТЕ И НОМЕНКЛАТУРАТА НА ЛЕКАРСТВЕНИТЕ ПРОДУКТИ*. Lex. Retrieved March 28, 2022, from <https://www.lex.bg/en/laws/ldoc/2135610889>

Bulgarian Drug Agency. (n.d.). *Нормативни актове*. Retrieved March 28, 2022, from <https://www.bda.bg/bg/%D0%BD%D0%BE%D1%80%D0%BC%D0%B0%D1%82%D0%B8%D0%B2%D0%BD%D0%B8-%D0%B0%D0%BA%D1%82%D0%BE%D0%B2%D0%B5>

National Council on Prices and Reimbursement of Medicinal Product. (2019, August 6). *ORDINANCE ON THE TERMS, RULES AND PROCEDURE FOR REGULATION AND REGISTRATION OF PRICES FOR MEDICINAL PRODUCTS*. Retrieved March 28, 2022, from <https://www.ncpr.bg/en/regulations/bulgarian-legislation/regulations/>

ИЗПЪЛНИТЕЛНА АГЕНЦИЯ ПО ЛЕКАРСТВАТА. (n.d.). *КРАТКИ ХАРАКТЕРИСТИКИ НА РАЗРЕШЕНИ ЗА УПОТРЕБА ЛЕКАРСТВЕНИ ПРОДУКТИ*. Bulgarian Drug Agency. Retrieved March 28, 2022, from <https://www.bda.bg/images/stories/documents/bdias/drugs2_list2_1.htm>

National Council on Prices and Reimbursement of Medicinal Products. (2022, May 2). *Drug search*. Retrieved May 17, 2022, from <https://portal.ncpr.bg/registers/pages/register/list-medicament.xhtml>

**Croatia**

Končić, M. Z., & Atkinson, J. (2018). Pharmacy Practice and Education in Croatia. *Pharmacy*, *6*(3), 89. <https://doi.org/10.3390/pharmacy6030089>

Jonjić, D., & Vitale, K. (2014). Issues around household pharmaceutical waste disposal through community pharmacies in Croatia. *International Journal of Clinical Pharmacy*, *36*(3), 556-563. <https://doi.org/10.1007/s11096-014-9936-7>

Agency for Medicinal Products and Medical Devices of Croatia. (n.d.). *Medicinal Products Database*. HALMED. Retrieved March 25, 2022, from <https://www.halmed.hr/en/Lijekovi/Baza-lijekova/>

Agency for Medicinal Products and Medical Devices of Croatia. (n.d.-b). *Over-the-counter (OTC) Medicines*. HALMED. Retrieved March 25, 2022, from <https://www.halmed.hr/en/Lijekovi/Informacije-o-lijekovima/Bezreceptni-OTC-lijekovi/>

Agencija za lijekove i medicinske proizvode. (n.d.). *Promet, proizvodnja i inspekcija - Krivotvoreni lijekovi*. HALMED. Retrieved March 25, 2022, from <https://www.halmed.hr/Promet-proizvodnja-i-inspekcija/Krivotvoreni-lijekovi/>

Employed Community Pharmacists in Europe (EPhEU). (2018, January 9). *More about pharmacy in Croatia*. Retrieved March 25, 2022, from <https://epheu.eu/croatia-more-about-pharmacy/#:%7E:text=The%20owner%20of%20an%20independent,30%20students>

Ministarstvo Zdravlja. (2014). *Pravilnik o uvjetima za davanje dozvole specijaliziranim prodavaonicama za promet na malo lijekovima*. Narodne Novine. Retrieved March 25, 2022, from <https://narodne-novine.nn.hr/clanci/sluzbeni/2014_10_122_2348.html>

Hrvatska Ljekarnička Komora. (1998). *Pravilnik o načinu oglašavanja rada ljekarnika privatne prakse i zdravstvenih ljekarničkih ustanova u privatnom vlasništvu*. Narodne Novine. Retrieved March 25, 2022, from <https://narodne-novine.nn.hr/clanci/sluzbeni/1998_05_66_800.html>

Ministarstvo zdravstva. (2018, November 22). *Zakon o lijekovima*. Zakon. Retrieved March 25, 2022, from <https://www.zakon.hr/z/399/Zakon-o-lijekovima>

**Cyprus**

Republic of Cyprus - Pharmaceutical Services, Ministry of Health Design & Development: Department of Information Technology Services. (n.d.). *Pharmacies - Online Pharmacies | Pharmaceutical Services |*. Ministry of Health Design & Development. Retrieved March 20, 2022, from <https://www.moh.gov.cy/moh/phs/phs.nsf/All/23DFAFA481BB9FEAC225857D0030E9B2?OpenDocument>

Republic of Cyprus - Pharmaceutical Services, Ministry of Health Design & Development: Department of Information Technology Services. (n.d.-b). *Pricing Policy | Pharmaceutical Services |*. Ministry of Health Design & Development. Retrieved April 20, 2022, from <https://www.moh.gov.cy/moh/phs/phs.nsf/pricingpolicy_en/pricingpolicy_en?OpenDocument>

Republic of Cyprus - Pharmaceutical Services, Ministry of Health Design & Development: Department of Information Technology Services. (n.d.). *Pharmacies - Pharmacies | Pharmaceutical Services |*. Ministry of Health Design & Development. Retrieved April 20, 2022, from <https://www.moh.gov.cy/moh/phs/phs.nsf/All/089F47BF3620A2CCC225857D002E0846?OpenDocument>

*Φαρμακεία - Επιχείρηση Φαρμακοποιού | Φαρμακευτικές Υπηρεσίες*. (n.d.). Retrieved April 20, 2022, from <https://www.moh.gov.cy/moh/phs/phs.nsf/All/A1D13D2E41A1AC64C225857A00314260?OpenDocument>

**Czech republic**

Nachtigal, P., Šimůnek, T., & Atkinson, J. (2017). Pharmacy practice and education in the Czech Republic. *Pharmacy*, *5*(4), 54. <https://doi.org/10.3390/pharmacy5040054>

AION CS. (2022, March 8). *228/2008 Sb. Vyhláška o registraci léčivých přípravků*. Zákony pro lidi. Retrieved April 13, 2022, from <https://www.zakonyprolidi.cz/nabidka/cs/2008-228/zneni-20080701#p20-1>

AION CS. (2022a, February 1). *378/2007 Sb. Zákon o léčivech*. Zákony pro lidi. Retrieved April 13, 2022, from <https://www.zakonyprolidi.cz/cs/2007-378>

AION CS. (2022a, January 1). *84/2008 Sb. Vyhláška o správné lékárenské praxi, bližších podmínkách zacházení s léčivy v lékárnách, zdravotnick. . .* Zákony pro lidi. Retrieved April 13, 2022, from <https://www.zakonyprolidi.cz/cs/2008-84>

SÚKL. (n.d.). *State Institute for Drug Control*. Retrieved April 14, 2022, from <https://www.sukl.eu/?sukl_session=r3jsgci294tohugfit8o1b6ka4>

SÚKL. (2022, April 30). *Medicinal products database*. Retrieved May 4, 2022, from <https://www.sukl.eu/modules/medication/search.php?lang=2>

SÚKL. (2021, April 16). *LEK-16 verze 4, Státní ústav pro kontrolu léčiv*. Retrieved April 14, 2022, from <https://www.sukl.cz/lekarny/lek-16-verze-4>

**Denmark**

Danish Medicines Agency. (2015, December 14). *Pharmacies and sale of medicines*. Retrieved March 29, 2022, from <https://laegemiddelstyrelsen.dk/en/pharmacies/>

Danish Medicines Agency. (2019, September 3). *Sale outside pharmacies*. Retrieved March 29, 2022, from <https://laegemiddelstyrelsen.dk/en/pharmacies/sale-outside-pharmacies/>

Danish Medicines Agency. (2019a, September 3). *Over-the-counter medicines*. Retrieved March 29, 2022, from <https://laegemiddelstyrelsen.dk/en/pharmacies/over-the-counter-medicines/>

Danish Medicines Agency. (2019a, July 1). *Prices of medicines*. Retrieved March 29, 2022, from <https://laegemiddelstyrelsen.dk/en/reimbursement/prices/>

Ministry of Health. (2017). *Healthcare in Denmark - an Overview.Version 1.2*. Ministry of Health. <https://www.healthcaredenmark.dk/media/ykedbhsl/healthcare-dk.pdf>

Sundheds- og Ældreministeriet. (2018, June 12). *LBK nr 801 af 12/06/2018 - Bekendtgørelse af lov om apoteksvirksomhed*. Retsinformation. Retrieved March 29, 2022, from <https://www.retsinformation.dk/eli/lta/2018/801>

Styrelsen for Patientsikkerhed. (n.d.). *Opbevare og bortskaffe medicin*. Retrieved March 29, 2022, from <https://stps.dk/da/ansvar-og-retningslinjer/vejledning/haandtering-af-medicin/korrekt-haandtering-af-medicin-uden-for-sygehusene/opbevare-og-bortskaffe-medicin/>

Lægemiddelstyrelsen. (n.d.). *Produktresuméer*. Produktresume. Retrieved March 29, 2022, from <http://www.produktresume.dk/AppBuilder/search>

**Estonia**

Sepp, K., Tuula, A., Bobrova, V., & Volmer, D. (2021). Primary health care policy and vision for community pharmacy and pharmacists in Estonia. *Pharmacy Practice (Granada)*, *19*(2). <http://doi.org/10.18549/PharmPract.2021.2.2404>

Gross, M., & Volmer, D. (2016). Restrictions to Pharmacy Ownership and Vertical Integration in Estonia—Perception of Different Stakeholders. *Pharmacy*, *4*(2), 18. <https://doi.org/10.3390/pharmacy4020018>

Konkurentsiamet. (2015, December 14). *New restrictions on establishment of pharmacies significantly harm free competition*. Retrieved February 20, 2022, from <https://www.konkurentsiamet.ee/en/news/new-restrictions-establishment-pharmacies-significantly-harm-free-competition>

Ravimiamet. (2022). *Tegevuslubade otsing*. Retrieved February 20, 2022, from <https://rkav.sm.ee/rkav/faces/pages/tegevuslubaForm/tegevuslubaOtsing.xhtml>

Ravimiamet. (2022b, January 4). *Apteek*. Retrieved February 20, 2022, from <https://www.ravimiamet.ee/ravimid-muugiload-ohutus-ja-kaitlemine/ravimi-kaitlemine/apteek>

Ravimiamet. (n.d.). *Ravimiregister*. Retrieved February 20, 2022, from <https://ravimiregister.ee/?pv=HumRavimid.Otsing>

Ravimiamet. (2022c, February 2). *Internetiapteek*. Retrieved February 20, 2022, from <https://ravimiamet.ee/ravimite-kaitlemine/ravimi-kaitlemine/internetiapteek>

**Finland**

*HE 295/2018 - Hallituksen esitykset*. (2018). FINLEX. Retrieved February 17, 2022, from <https://www.finlex.fi/fi/esitykset/he/2018/20180295#idm45237815573088>

*Buying medicines online*. (2020, May 27). EU-Healthcare.Fi. Retrieved February 17, 2022, from https://www.eu-healthcare.fi/medicines/medicines-purchased-online/

*Ordering medicines online*. (n.d.). FIMEA. Retrieved February 17, 2022, from <https://www.fimea.fi/web/en/for_public/internet-trade-in-medicines>

Katja Lösönen, U. O. (2021, May 10). *Kuka päättää lääkkeen hinnan?* Apteekki. Retrieved February 17, 2022, from <https://www.apteekki.fi/terveydeksi/laaketietoa/kuka-paattaa-laakkeen-hinnan.html>

*Valtioneuvoston asetus lääketaksasta 713/2013 - Ajantasainen lainsäädäntö*. (2022, March 24). FINLEX. Retrieved May 17, 2022, from https://www.finlex.fi/fi/laki/ajantasa/2013/20130713#P4

*Lääkehaku*. (n.d.). Apteekki. Retrieved February 17, 2022, from <https://www.apteekki.fi/laakehaku.html>

FIMEA. (n.d.). *FimeaWeb*. Retrieved February 17, 2022, from <https://www.fimea.fi/web/en/databases_and_registers/fimeaweb>

**France**

Ministère des Solidarités et de la Santé. (2022, March 16). *Le circuit de distribution du médicament en France*. Retrieved January 17, 2022, from <https://solidarites-sante.gouv.fr/soins-et-maladies/medicaments/le-circuit-du-medicament/article/le-circuit-de-distribution-du-medicament-en-france>

Ordre National Des Pharmacies. (2021, May 19). *Vente de médicaments sur Internet en France - Les patients - Ordre National des Pharmaciens*. Retrieved January 17, 2022, from <http://www.ordre.pharmacien.fr/Les-patients/Vente-de-medicaments-sur-Internet-en-France>

*Code de la santé publique - Article L5121-5*. (2016, July 15). Légifrance (Gouvernement Français). Retrieved January 17, 2022, from <https://www.legifrance.gouv.fr/codes/article_lc/LEGIARTI000032906195/>

*Code de la santé publique - Article L5125-33*. (2020, December 9). Légifrance (Gouvernement Français). Retrieved January 17, 2022, from <https://www.legifrance.gouv.fr/codes/article_lc/LEGIARTI000042655996/>

*Code de la santé publique - Article R5125-70*. (2015, July 1). Légifrance (Gouvernement Français). Retrieved January 17, 2022, from <https://www.legifrance.gouv.fr/codes/article_lc/LEGIARTI000026897520/>

Ministère des Solidarités et de la Santé. (2016, June 13). *Prix et tarif des médicaments (spécialités pharmaceutiques)*. Retrieved January 17, 2022, from <https://solidarites-sante.gouv.fr/soins-et-maladies/medicaments/professionnels-de-sante/consulter-la-reglementation-sur-les-medicaments/article/prix-et-tarif-des-medicaments-specialites-pharmaceutiques>

*Code de la santé publique - Chapitre V : Pharmacie d’officine. Section 2: General conditions of authorization (Articles L5125-3 to L5125-5-1)*. (2018, January 3). Légifrance (Gouvernement Français). Retrieved January 17, 2022, from <https://www.legifrance.gouv.fr/codes/id/LEGISCTA000036401035/2018-07-31>

*Code de la santé publique - Chapitre V : Pharmacie d’officine. (Articles L5125-1 à L5125-22)*. (2009, July 21). Légifrance (Gouvernement Français). Retrieved January 17, 2022, from <https://www.legifrance.gouv.fr/codes/id/LEGISCTA000020890194/2009-07-23>

Agence Nationale de Sécurité du Médicament et des Produits de Santé (ANSM). (2022, April 29). *Autorisation - Accueil*. ANSM. Retrieved May 17, 2022, from <http://agence-prd.ansm.sante.fr/php/ecodex/index.php>

**Germany**

Bundesministerium für Gesundheit. (2021, March 26). *Preisgestaltung für nicht verschreibungspflichtige Arzneimittel*. Retrieved January 28, 2022, from <https://www.bundesgesundheitsministerium.de/themen/krankenversicherung/online-ratgeber-krankenversicherung/arznei-heil-und-hilfsmittel/preisgestaltung-fuer-nicht-verschreibungspflichtige-arzneimittel.html>

Bundesministerium für Gesundheit. (2020, October 21). *Verordnung über apothekenpflichtige und freiverkäufliche Arzneimittel*. Bundesministerium Der Justiz. Retrieved January 28, 2022, from <https://www.gesetze-im-internet.de/amverkrv/BJNR021050988.html>

ABDA. (n.d.). *Versandhandel*. Retrieved January 28, 2022, from <https://www.abda.de/themen/versorgungsfragen/versandhandel/>

ABDA. (n.d.-a). *Grundlagen für den Apothekenbetrieb*. Retrieved January 28, 2022, from <https://www.abda.de/apotheke-in-deutschland/grundlagen-fuer-den-apothekenbetrieb/>

Bundesministerium für Gesundheit. (2021b, September 27). *Gesetz über den Verkehr mit Arzneimitteln (Arzneimittelgesetz - AMG)*. Bundesministerium Der Justiz. Retrieved January 28, 2022, from <https://www.gesetze-im-internet.de/amg_1976/BJNR024480976.html>

Bundesinstitut für Arzneimittel und Medizinprodukte (BfArM). (n.d.). *PharmNet.Bund - Drug Information System*. PharmNet. Retrieved January 28, 2022, from <https://www.pharmnet-bund.de/dynamic/en/drug-information-system/index.html>

Paris, V. and E. Docteur (2007), "Pharmaceutical pricing and reimbursement policies in Germany.", *OECD Health Working Papers*, No. 39, OECD Publishing, Paris. <http://dx.doi.org/10.2139/ssrn.1320147>

**Greece**

Kalavrezou, N. and Jin, H., (2021) Healthcare Reform in Greece: Progress and Reform Priorities. *IMF Working Papers*, *2021*(189). <https://doi.org/10.5089/9781513588834.001>

Yfantopoulos, J. N., & Chantzaras, A. (2018). Drug policy in Greece. *Value in Health Regional Issues*, *16*, 66-73. <https://doi.org/10.1016/j.vhri.2018.06.006>

Government Gazette. (2017, May 29). *ΦΕΚ 1761/Β/22.05.2017- Καθορισμος ενδεικτικής τιμής λιανικής πώλησης & ανώτατης νοσοκομειακής τιμής – ΜΗ.ΣΥ.ΦΑ.* SFEE. Retrieved February 23, 2022, from <https://www.sfee.gr/fek-1761v22-05-2017-kathorismos-endiktikis-timis-lianikis-polisis-anotatis-nosokomiakis-timis-mi-si-fa/>

Government Gazette. (2016, May 30). *ΦΕΚ 1445/Β/23.06.2016 – Ρυθμίσεις επαγγέλματος φαρμακοποιού – Ίδρυση φαρμακείου*. SFEE. Retrieved February 23, 2022, from <https://www.sfee.gr/fek-1445v23-06-2016-rithmisis-epangelmatos-farmakopiou-idrisi-farmakiou/>

Government Gazette. (2017a, May 22). *ΦΕΚ 2219/Β/18.07.2016 – Διάθεση ΓΕΔΙΦΑ*. SFEE. Retrieved February 23, 2022, from <https://www.sfee.gr/fek-2219v18-07-2016-diathesi-gedifa/>

Government Gazette. (2018, July 11). *ΦΕΚ 124/Α/11.7.2018 - Ρυθμίσεις επαγγέλματος φαρμακοποιού - Ίδρυση φαρμακείου.* Τράπεζα Πληροφοριών Νομοθεσίας. Retrieved February 23, 2022, from <https://www.e-nomothesia.gr/kat-ygeia/farmakeia/proedriko-diatagma-64-2018-phek-124a-11-7-2018.html>

Υπουργείου Υγείας. (2016, May 18). *Δημιουργία της υποκατηγορίας «Γενικής Διάθεσης Φάρμακα» (ΓΕΔΙΦΑ)*. Retrieved February 23, 2022, from <https://www.moh.gov.gr/articles/ministry/grafeio-typoy/press-releases/3917>

ΕΟΠΥΥ*.* (n.d.). *Ε.Ο.Π.Υ.Υ. - Εθνικός Οργανισμός Παροχής Υπηρεσιών Υγείας*. Retrieved February 23, 2022, from https://www.eopyy.gov.gr/medicine/list

Arvanitis, C. (2014, April 10). *Ερμηνεία του Νέου Νόμου για τα Φαρμακεία*. Χρήστος Δ. Αρβανίτης & Συνεργάτες. Retrieved February 23, 2022, from <https://arvanitislaw.gr/blog/2014/04/10/%CE%B5%CF%81%CE%BC%CE%B7%CE%BD%CE%B5%CE%AF%CE%B1-%CF%84%CE%BF%CF%85-%CE%BD%CE%AD%CE%BF%CF%85-%CE%BD%CF%8C%CE%BC%CE%BF%CF%85-%CE%B3%CE%B9%CE%B1-%CF%84%CE%B1-%CF%86%CE%B1%CF%81%CE%BC%CE%B1%CE%BA%CE%B5/>

Government Gazette. (2014, April 7). *ΦΕΚ Α 85/07.04.2014 - Μέτρα στήριξης και ανάπτυξης της ελληνικής οικονομίας στο πλαίσιο εφαρμογής του ν. 4046/2012 και άλλες διατάξεις.* kodiko.gr. Retrieved February 23, 2022, from <https://www.kodiko.gr/nomothesia/document/96016/nomos-4254-2014>

**Hungary**

National Institute of Pharmacy and Nutrition. (2022, April 21). *Lists*. Retrieved May 16, 2022, from <https://ogyei.gov.hu/lists>

National Institute of Pharmacy and Nutrition. (2019, June 12). *Laws*. Retrieved May 16, 2022, from <https://ogyei.gov.hu/laws_and_regulations>

National Institute of Pharmacy and Nutrition. (2018, November 28). *Gyógyszertáron kívüli gyógyszerforgalmazás*. Retrieved May 16, 2022, from <https://ogyei.gov.hu/gyogyszertaron_kivuli_gyogyszerforgalmazas>

National Institute of Pharmacy and Nutrition. (2021, September 7). *Magyar jogszabályok*. Retrieved May 16, 2022, from <https://ogyei.gov.hu/magyar_jogszabalyok>

National Institute of Pharmacy and Nutrition. (2021b, November 18). *OGYÉI állásfoglalások*. Retrieved May 16, 2022, from <https://ogyei.gov.hu/ogyei_allasfoglalasok>

National Institute of Pharmacy and Nutrition. (2020, June 3). *Internetes gyógyszer-kereskedelem*. Retrieved May 16, 2022, from <https://ogyei.gov.hu/internetes_gyogyszer_kereskedelem/>

Wolter Kluwer. (2005). *52/2005. (XI. 18.) EüM rendelet az emberi alkalmazásra kerülő gyógyszerek forgalomba hozataláról - Hatályos Jogszabályok Gyűjteménye*. Wolters Kluger. Retrieved May 16, 2022, from <https://net.jogtar.hu/jogszabaly?docid=a0500052.eum#lbj196id3495>

National Institute of Pharmacy and Nutrition. (n.d.). *Gyógyszer-adatbázis*. Retrieved May 16, 2022, from <https://ogyei.gov.hu/gyogyszeradatbazis/>

**Ireland**

Gorecki, P. K. (2011). Do you believe in magic? Improving the quality of pharmacy services through restricting entry and aspirational contracts, the Irish experience. *The European Journal of Health Economics*, *12*(6), 521-531. <https://doi.org/10.1007/s10198-010-0264-0>

Health Products Regulatory Authority (HPRA). (n.d.). *Retailers and General Sale Medicines*. Retrieved February 3, 2022, from <http://www.hpra.ie/homepage/medicines/regulatory-information/retailers>

Pharmaceutical Society of Ireland (PSI). (n.d.). *Internet Supply Medicines*. Retrieved February 3, 2022, from <https://www.thepsi.ie/gns/Internet-Supply/Internet_supply_list_overview.aspx#:%7E:text=In%20Ireland%2C%20only%20non%2Dprescription,allowed%20to%20be%20sold%20online.&text=General%20sales%20medicines%3A%20These%20are,such%20as%20supermarkets%20and%20newsagents>

Pharmaceutical Society of Ireland (PSI). (n.d.-a). *Guidelines for Pharmacies*. Retrieved February 3, 2022, from <https://www.thepsi.ie/gns/Pharmacy_Practice/practice-guidance/Guidelines_for_Pharmacies.aspx>

Houses of the Oireachtas. (2020, June 3). *Medicinal Products*. Retrieved February 3, 2022, from <https://www.oireachtas.ie/en/debates/question/2020-06-03/694/>

Department of Health. (1996, May 30). *S.I. No. 152/1996 - Health (Community Pharmacy Contractor Agreement) Regulations, 1996*. Electronic Irish Statute Book (eISB). Retrieved February 3, 2022, from <https://www.irishstatutebook.ie/eli/1996/si/152/made/en/print>

Health Products Regulatory Authority (HPRA). (n.d.-a). *Find a medicine*. Retrieved February 3, 2022, from <http://www.hpra.ie/homepage/medicines/medicines-information/find-a-medicine>

**Italy**

Gallone, E. L., Enri, L. R., Pignata, I., Baratta, F., & Brusa, P. (2020). The 2017 deregulation of pharmacies in Italy: Introducing non-pharmacist ownership. *Health Policy*, *124*(12), 1281-1286. <https://doi.org/10.1016/j.healthpol.2020.08.013>

*Gazzetta Ufficiale - PLEGGE 2 aprile 1968, n. 475*. (1968, April 27). Retrieved January 20, 2022, from <https://www.gazzettaufficiale.it/atto/serie_generale/caricaDettaglioAtto/originario?atto.dataPubblicazioneGazzetta=1968-04-27>

Ministero della Salute. (2015, June 23). *Vendita on line di medicinali senza obbligo di prescrizione, precisazioni del Ministero*. Retrieved January 20, 2022, from <https://www.salute.gov.it/portale/news/p3_2_1_1_1.jsp?lingua=italiano&menu=notizie&p=null&id=2133>

Ministero della Salute. (2014, February 19). *Attuazione della direttiva 2011/62/UE, che modifica la direttiva 2001/83/CE, recante un codice comunitario relativo ai medicinali per uso umano, al fine di impedire l’ingresso di medicinali falsificati nella catena di fornitura legale*. Trova Norme & Concorsi - Normativa Sanitaria. Retrieved January 20, 2022, from <https://www.trovanorme.salute.gov.it/norme/dettaglioAtto?id=48428>

Ministero della Salute. (2006, August 4). *Disposizioni urgenti per il rilancio economico e sociale, per il contenimento e la razionalizzazione della spesa pubblica, nonche’ interventi in materia di entrate e di contrasto all’evasione fiscale*. Trova Norme & Concorsi - Normativa Sanitaria. Retrieved January 20, 2022, from <https://www.trovanorme.salute.gov.it/norme/dettaglioAtto?id=29585>

Ministero della Salute. (2019, April 30). *Esercizi commerciali (Parafarmacie)*. Retrieved January 20, 2022, from <https://www.salute.gov.it/portale/tracciabilita/dettaglioContenutiTracciabilita.jsp?lingua=italiano&id=5092&area=tracciabilita%20farmaco&menu=produzioneDistribuzione>

Ministero della Salute. (1998, April 24). *Riforma della disciplina relativa al settore del commercio, a norma dell’articolo 4, comma 4, della legge 15 marzo 1997, n. 59*. Trova Norme & Concorsi - Normativa Sanitaria. Retrieved January 20, 2022, from <https://www.trovanorme.salute.gov.it/norme/dettaglioAtto?id=23960&articolo=4>

Ministero della Salute. (2011, December 22). *Conversione in legge, con modificazioni, del decreto-legge 6 dicembre 2011, n. 201, recante disposizioni urgenti per la crescita, l’equita’ e il consolidamento dei conti pubblici*. Trova Norme & Concorsi - Normativa Sanitaria. Retrieved January 20, 2022, from <https://www.trovanorme.salute.gov.it/norme/dettaglioAtto?id=41148&completo=true>

Ministero della Salute. (2021, July 6). *Trasparenza sui SOP*. Retrieved January 20, 2022, from <https://www.salute.gov.it/portale/tracciabilita/dettaglioContenutiTracciabilita.jsp?lingua=italiano&id=5165&area=tracciabilita%20farmaco&menu=vuoto>

Ministero della Salute. (1991, November 16). *Gazzetta Ufficiale - LEGGE 8 novembre 1991, n. 362*. Gazzetta Ufficiale. Retrieved January 20, 2022, from <https://www.gazzettaufficiale.it/atto/serie_generale/caricaDettaglioAtto/originario?atto.dataPubblicazioneGazzetta=1991-11-16&atto.codiceRedazionale=091G0244&elenco30giorni=false>

Agenzia Italiana del Farmaco. (n.d.). *Home | Banca Dati Farmaci dell’AIFA*. Retrieved January 20, 2022, from <https://farmaci.agenziafarmaco.gov.it/bancadatifarmaci/home>

**Latvia**

Muceniece, R., Riekstina, U., Maurina, B., Enina, V., & Atkinson, J. (2018). Pharmacy practice and education in Latvia. *Pharmacy*, *6*(1), 9. <https://doi.org/10.3390/pharmacy6010009>

Silins, J., & Szkultecka-Dębek, M. (2017). Drug policy in Latvia. *Value in health regional issues*, *13*, 73-78. <https://doi.org/10.1016/j.vhri.2017.08.006>

Cabinet of Ministers. (2016, May 6). *Criteria for the Location of Pharmacies and Pharmacy Branches*. LIKUMI. Retrieved January 11, 2022, from <https://likumi.lv/ta/en/en/id/234279>

State Agency of Medicines of the Republic of Latvia. (n.d.). *Purchase of medicines in pharmacy*. Retrieved January 11, 2022, from <https://www.zva.gov.lv/en/patients-and-public/medicines/purchase-medicines-pharmacy>

State Agency of Medicines of the Republic of Latvia. (n.d.-a). *Online pharmacies*. Retrieved January 11, 2022, from <https://www.zva.gov.lv/en/patients-and-public/medicines/purchase-medicines-pharmacy/online-pharmacies>

State Agency of Medicines of the Republic of Latvia. (n.d.-b). *Publications*. Retrieved January 11, 2022, from <https://www.zva.gov.lv/en/publications>

Latvijas Vēstnesis. (n.d.). *Legal acts of the Republic of Latvia*. Likumi. Retrieved January 11, 2022, from <https://likumi.lv/about.php>

Cabinet of Ministers. (2015, August 27). *Regulations Regarding the Principles for the Determination of the Price of Medicinal Products*. LIKUMI. Retrieved January 11, 2022, from <https://likumi.lv/ta/en/en/id/120074-regulations-regarding-the-principles-for-the-determination-of-the-price-of-medicinal-products>

State Agency of Medicines of the Republic of Latvia. (n.d.-a). *Medicinal Product Register of Latvia*. Retrieved January 11, 2022, from <https://dati.zva.gov.lv/zalu-registrs/?&lang=en>

**Lithuania**

State Medicines Control Agency of Lithuania. (n.d.). *Legislation*. Retrieved February 8, 2022, from <https://www.vvkt.lt/index.php?1148175238>

Lietuvos Respublikos Seimas. (2022, January 31). *X-709 Lietuvos Respublikos farmacijos Ä¯statymas*. E-Seimas. Retrieved February 8, 2022, from <https://e-seimas.lrs.lt/portal/legalAct/lt/TAD/TAIS.280067>

Lietuvos Respublikos sveikatos apsaugos ministerija. (2020, May 19). *Jau greitai receptinius vaistus bus galima įsigyti ir internetu*. Retrieved February 8, 2022, from <https://sam.lrv.lt/lt/naujienos/jau-greitai-receptinius-vaistus-bus-galima-isigyti-ir-internetu>

Lietuvos Respublikos Seimas. (2003). *ĮSTATYMAS FARMACINĖS VEIKLOS ĮSTATYMO PAKEITIMO*. Retrieved February 8, 2022, from <https://e-seimas.lrs.lt/portal/legalActPrint/lt?jfwid=-9dzqnub28&documentId=TAIS.225492&category=TAP>

Losinskij, B. (2021). Visuomenės vaistinių pacientų nuomonė apie nereceptinių vaistų įsigijimą mažmeninėse prekybos įmonėse. Available in https://www.lsmu.lt/cris/handle/20.500.12512/111214

Valstybinė vaistų kontrolės tarnyba. (n.d.). *Medicines search*. Retrieved February 8, 2022, from <https://vapris.vvkt.lt/vvkt-web/public/medications?lang=en>

Lietuvos Respublikos Seimas. (2019, September 2). *Nr. (1.72E)1A-1567 - Dėl Vaistinių preparatų, leidžiamų parduoti vaistinių preparatų mažmeninės prekybos įmonėse, sąrašo patvirtinimo pakeitimo*. Retrieved February 8, 2022, from <https://www.e-tar.lt/portal/lt/legalAct/1b2d6870cd7111e9929af1b9eea48566>

*V-203 DÄl Nekompensuojamojo vaistinio preparato registruotojo ar lygiagretaus importo leidimo turÄtojo . . .* (n.d.). Retrieved January 11, 2022, from <https://www.e-tar.lt/portal/lt/legalAct/TAR.A8E42F1FE02D>

**Luxembourg**

Rémuzat, C., Urbinati, D., Mzoughi, O., El Hammi, E., Belgaied, W., & Toumi, M. (2015). Overview of external reference pricing systems in Europe. *Journal of market access & health policy*, *3*(1), 27675. <https://doi.org/10.3402/jmahp.v3.27675>

Journal Officiel du Grand-Duché de Luxembourg. (1997, May 27). *Règlement grand-ducal du 27 mai 1997 relatif à l’octroi des concessions de pharmacie. - Legilux*. Retrieved February 12, 2022, from <https://legilux.public.lu/eli/etat/leg/rgd/1997/05/27/n1/jo>

Journal Officiel du Grand-Duché de Luxembourg. (2002, February 11). *Règlement grand-ducal du 11 février 2002 modifiant le règlement grand-ducal du 27 mai 1997 relatif à l’octroi des concessions de pharmacie. - Legilux*. Retrieved February 12, 2022, from <https://legilux.public.lu/eli/etat/leg/rgd/2002/02/11/n2/jo>

Journal Officiel du Grand-Duché de Luxembourg. (2013, October 18). *Règlement grand-ducal du 18 octobre 2013 modifiant le règlement grand-ducal modifié du 27 mai 1997 relatif à l’octroi des concessions de pharmacie. - Legilux*. Retrieved February 12, 2022, from <https://legilux.public.lu/eli/etat/leg/rgd/2013/10/18/n3/jo>

Ordre National des Pharmaciens. (n.d.). *Ordre National des Pharmaciens*. Retrieved February 12, 2022, from <http://www.ordre.pharmacien.fr/>

Journal Officiel du Grand-Duché de Luxembourg. (2011, December 1). *Règlement grand-ducal du 1er décembre 2011 déterminant les critères, les conditions et la procédure relative à la fixation des prix des médicaments à usage humain. - Legilux*. Retrieved February 12, 2022, from <https://legilux.public.lu/eli/etat/leg/rgd/2011/12/01/n5/jo>

Institutions de Sécurité Sociale. (2011, December 1). *Règlement grand-ducal modifié du 1er décembre 2011 - CODE DE LA SÉCURITÉ SOCIALE*. Retrieved February 12, 2022, from <https://www.secu.lu/assurance-maladie/reglements/reglement-grand-ducal-modifie-du-1er-decembre-2011/>

Institutions de Sécurité Sociale. (n.d.). *Prestations de soins de santé - CODE DE LA SÉCURITÉ SOCIALE*. Retrieved February 12, 2022, from <https://www.secu.lu/assurance-maladie/livre-i/chapitre-ii-objet-de-lassurance/prestations-de-soins-de-sante/art-22/>

Journal Officiel du Grand-Duché de Luxembourg. (2018, August 16). *Règlement grand-ducal du 18 juillet 2018 relatif à la préparation, à la division, au conditionnement ou reconditionnement et à la vente par internet de médicaments*. Retrieved February 12, 2022, from <https://legilux.public.lu/eli/etat/leg/rgd/2018/07/18/a683/jo>

**Malta**

Government of Malta. (n.d.). *Legal Notice 279 of 2007 - Pharmacy Licence Regulations, 2007*. Legislation Malta. Retrieved April 18, 2022, from <https://legislation.mt/eli/ln/2007/279/eng/pdf#:~:text=(1)%20The%20number%20of%20pharmacies,pharmacies%20already%20holding%20a%20licence>

Government of Malta. (2020, July 14). *CHAPTER 458 - MEDICINES ACT*. Legislation Malta. Retrieved April 18, 2022, from <https://legislation.mt/eli/cap/458/eng/pdf>

Malta Medicines Authority. (n.d.). *Buying Medicinal Products Over The Internet And Internet Pharmacies*. Retrieved April 19, 2022, from <https://medicinesauthority.gov.mt/internetpharmacies>

Government of Malta. (2007, October 5). *SUBSIDIARY LEGISLATION 458.16 - PHARMACY LICENCE REGULATIONS*. Legislation Malta. Retrieved April 18, 2022, from <https://legislation.mt/eli/sl/458.16/eng/pdf>

Medicines Malta Authority. (n.d.). *Home*. Retrieved April 19, 2022, from <https://medicinesauthority.gov.mt/>

**Netherlands**

Ministerie van Volksgezondheid, Welzijn en Sport. (2022, January 31). *Regeling Geneesmiddelenwet*. Overheid. Retrieved February 4, 2022, from <https://wetten.overheid.nl/BWBR0022160/2022-01-31/0>

College ter Beoordeling van Geneesmiddelen. (2021, September 1). *Medicijnen met en zonder recept*. Retrieved February 4, 2022, from <https://www.cbg-meb.nl/onderwerpen/medicijninformatie-medicijnen-met-en-zonder-recept#modal-5>

Ministerie van Algemene Zaken. (2015, September 29). *What do I pay for prescription drugs?* Government of Netherlands. Retrieved February 4, 2022, from <https://www.government.nl/topics/medicines/question-and-answer/what-do-i-pay-for-prescription-drugs>

College ter Beoordeling van Geneesmiddelen. (2021a, January 22). *Legal status of supply*. Medicines Evaluation Board. Retrieved February 4, 2022, from <https://english.cbg-meb.nl/topics/mah-legal-status-of-supply-and-otc-medicinal-products>

College ter Beoordeling van Geneesmiddelen. (2021b, July 7). *GS legal status of supply*. Medicines Evaluation Board. Retrieved February 4, 2022, from <https://english.cbg-meb.nl/topics/mah-legal-status-of-supply-and-otc-medicinal-products/gs-legal-status-of-supply>

College ter Beoordeling van Geneesmiddelen. (2022, May 11). *Geneesmiddeleninformatiebank*. Medicines Evaluation Board. Retrieved May 16, 2022, from <https://www.geneesmiddeleninformatiebank.nl/ords/f?p=111:1:0::NO:RP,1:P0_DOMAIN,P0_LANG:H,EN>

**Norway**

Statens Legemiddelverk. (2018, April 16). *Generelt om pris på legemidler*. Retrieved February 9, 2022, from <https://legemiddelverket.no/offentlig-finansiering/pris-pa-legemidler#priser-p%C3%A5-reseptfrie-legemidler>

Statens Legemiddelverk. (2018a, March 26). *Liste over legemidler som kan omsettes utenfor apotek*. Retrieved February 9, 2022, from <https://legemiddelverket.no/import-og-salg/salg-utenom-apotek/liste-over-legemidler-som-kan-omsettes-utenfor-apotek>

Statens Legemiddelverk. (2022, January 17). *Veiledning til utsalgssteder som skal selge legemidler utenom apotek*. Retrieved February 9, 2022, from <https://legemiddelverket.no/import-og-salg/salg-utenom-apotek/veiledning-til-utsalgssteder-som-skal-selge-legemidler-utenom-apotek#er-det-mengdebegrensninger-ved-salg-av-legemidler?->

Helse-og omsorgsdepartementet. (2022, January 1). *Lov om apotek (apotekloven)*. Lovdata. Retrieved February 9, 2022, from <https://lovdata.no/dokument/NL/lov/2000-06-02-39>

Statens Legemiddelverk. (2021, September 10). *Tillatelse til å eie og drive apotek*. Retrieved February 9, 2022, from <https://legemiddelverket.no/import-og-salg/apotekdrift/apotektillatelser#s%C3%B8knad-om-apotekkonsesjon>

Statens Legemiddelverk. (2022b, April 8). *Godkjente nettapotek og andre registrerte utsalgssteder for salg av legemidler på nett*. Retrieved May 15, 2022, from <https://legemiddelverket.no/import-og-salg/apotekdrift/registreringsordning-for-netthandel-med-legemidler/godkjente-utsalgssteder>

Statens Legemiddelverk. (n.d.). *Legemidler som utleveres uten resept - OTC*. Retrieved February 9, 2022, from <https://legemiddelverket.no/godkjenning/godkjenning-av-legemidler/reseptfrihet-otc>

Statens Legemiddelverk. (n.d.-a). *Legemiddelsøk*. Retrieved February 10, 2022, from <https://www.legemiddelsok.no/>

Statens Legemiddelverk. (2010, December 14). *Prisundersøkelse 2010 LUA-ordningen*. Retrieved February 10, 2022, from <https://legemiddelverket.no/Documents/Offentlig%20finansiering%20og%20pris/Pris/Pris%20p%C3%A5%20reseptfrie%20legemidler/prisunders%C3%B8kelse_2010.pdf>

**Poland**

Wiśniewski, M., Religioni, U., & Merks, P. (2020). Community Pharmacies in Poland—The Journey from a Deregulated to a Strictly Regulated Market. *International Journal of Environmental Research and Public Health*, *17*(23), 8751. <https://doi.org/10.3390/ijerph17238751>

Chief Pharmaceutical Inspectorate. (n.d.). *Online medicines sale*. Republic of Poland. Retrieved February 21, 2022, from <https://www.gov.pl/web/chief-pharmaceutical-inspectorate/online-medicines-sale>

*RAPORT Pozaapteczny obrót lekami OTC*. (n.d.). Stowarzyszenie Leki Tylko z Apteki. Retrieved February 21, 2022, from <https://lekitylkozapteki.pl/strona/raport-pozaapteczny-obrot-lekami-otc>

*Dziennik Ustaw - RZECZYPOSPOLITEJ POLSKIEJ*. (n.d.). Dziennik Ustaw - RZECZYPOSPOLITEJ POLSKIEJ. Retrieved February 21, 2022, from <https://dziennikustaw.gov.pl/DU>

*Poz. 208 ROZPORZĄDZENIE w sprawie wykazu substancji czynnych wchodzących w skład produktów leczniczych, które mogą być dopuszczone do obrotu w placówkach obrotu pozaaptecznego oraz punktach aptecznych, oraz kryteriów klasyfikacji tych produktów do poszczególnych wykazów*. (2022, January 28). Dziennik Ustaw - RZECZYPOSPOLITEJ POLSKIEJ. Retrieved February 21, 2022, from <https://dziennikustaw.gov.pl/D2022000020801.pdf>

Luty, O. (2010, March 28). *Sprzedaż leków małoletnim*. Domański Zakrzewski Palinka (DZP). Retrieved February 21, 2022, from <https://www.dzp.pl/blog/pharma/sprzedaz-lekow-maloletnim/>

Celejewski, B. (2021, November 8). *Obrót produktami leczniczymi*. Ministerstwo Zdrowia. Retrieved February 21, 2022, from <https://www.gov.pl/web/zdrowie/obrot-produktami-leczniczymi1>

Naczelna Izba Aptekarska. (2017, June 26). *NOWELIZACJA USTAWY – PRAWO FARMACEUTYCZNE (TZW. „APTEKA DLA APTEKARZA”)*. Retrieved February 21, 2022, from <https://www.nia.org.pl/2017/06/26/nowelizacja-ustawy-prawo-farmaceutyczne-tzw-apteka-dla-aptekarza/>

Rejestru Produktów Leczniczych (RPL). (n.d.). *Znajdź produkt leczniczy*. Retrieved February 21, 2022, from <https://rejestrymedyczne.ezdrowie.gov.pl/rpl/search/public>

**Portugal**

Moura, A., & Barros, P. P. (2020). Entry and price competition in the over‐the‐counter drug market after deregulation: Evidence from Portugal. *Health economics*, *29*(8), 865-877. <https://doi.org/10.1002/hec.4109>

Autoridad Nacional Do Medicamento e Productos de saúde. (2022). *Lista de locais de venda MNSRM*. INFARMED. Retrieved January 14, 2022, from <https://www.infarmed.pt/web/infarmed/entidades/licenciamentos/locais-de-venda-mnsrm/lista-de-locais-de-venda-mnsrm>

Autoridad Nacional Do Medicamento e Productos de saúde. (n.d.). *Locais de venda MNSRM*. INFARMED. Retrieved January 14, 2022, from <https://www.infarmed.pt/web/infarmed/entidades/licenciamentos/locais-de-venda-de-medicamentos-nao-sujeitos-a-receita-medica-lvmnsrm>

Ministério da Saúde. (2015, June 1). *Diário da República n.^o^ 105/2015, Série I de 2015–06-01, páginas 3453 - 3464*. Diário Da República Electrónico. Retrieved January 14, 2022, from <https://dre.pt/dre/detalhe/decreto-lei/97-2015-67356991>

Casanova, M. A. R. (2011). Medicamentos Não Sujeitos a Receita Médica (MNSRM). *Requisitos regulamentares e análise da evolução do mercado. Colégio da especialidade de assuntos regulamentares Ordem dos Farmacêuticos*. Retrieved January 14, 2022, from <https://www.ordemfarmaceuticos.pt/fotos/editor2/Colegios_de_Especialidade/Titulo_Especialidade/Especialidade_AR/Especialistas_Anteriores/2011/2011_Maria_Angels_Rafel_Casanova.pdf>

Assembleia da República. (2005, June 21). *Lei 38/2005, de 21 de Junho*. Diários da República. Retrieved January 14, 2022, from <https://dre.tretas.org/dre/186920/lei-38-2005-de-21-de-junho>

Assembleia da República. (2005b, August 16). *Decreto-lei 134/2005, de 16 de Agosto*. Diários da República. Retrieved January 14, 2022, from <https://dre.tretas.org/dre/188658/decreto-lei-134-2005-de-16-de-agosto>

Ministério da Saúde. (2022, January 14). *Decreto-Lei n.^o^ 307/2007 - O regime jurídico das farmácias de oficina*. Diário Da República Electrónico. Retrieved January 14, 2022, from <https://dre.pt/dre/legislacao-consolidada/decreto-lei/2007-75425909>

Ministério da Saúde. (2012, October 30). *Portaria n.^o^ 352/2012, de 30 de outubro*. Diário Da República Electrónico. Retrieved January 14, 2022, from <https://dre.pt/dre/detalhe/portaria/352-2012-191539>

Autoridad Nacional Do Medicamento e Productos de saúde. (n.d.-a). *Dispensa de medicamentos ao domicílio ou através da Internet*. INFARMED. Retrieved January 14, 2022, from <https://www.infarmed.pt/web/infarmed/entidades/licenciamentos/farmacias/servicos-aos-utentes/dispensa_domicilio_internet>

Instituto Nacional da Farmácia e do Medicamento (INFARMED). (2005, December 7). *Deliberação n.^o^ 1706/2005, de 7 de Dezembro - Registo prévio dos pontos de venda de medicamentos não sujeitos a receita médica*. INFARMED. Retrieved January 14, 2022, from <https://www.infarmed.pt/documents/15786/1068384/035-D_Delib_1706_2005_VF.pdf>

Instituto Nacional da Farmácia e do Medicamento (INFARMED). (2005a, September 14). *Portaria n.^o^ 827/2005, de 14 de Setembro - Estabelece as condições de venda de medicamentos não sujeitos a receita médica (MNSRM)*. INFARMED. Retrieved January 14, 2022, from <https://www.infarmed.pt/documents/15786/17838/portaria_827-2005.pdf/616667b1-92e0-437f-b411-ca71abcf5369>

Ministério da Saúde. (2005, August 16). *Decreto-Lei n.o 134/2005 de 16 de Agosto*. Diário Da República. Retrieved January 14, 2022, from <https://files.dre.pt/1s/2005/08/156a00/47634765.pdf>

Ministério da Saúde. (2007, June 19). *Decreto-Lei n.o 238/2007 de 19 de Junho*. Diário Da República. Retrieved January 14, 2022, from <https://files.dre.pt/1s/2007/06/11600/38983900.pdf>

INFOMED. (n.d.). *Base de dados de medicamentos de uso humano*. Retrieved January 14, 2022, from <https://extranet.infarmed.pt/INFOMED-fo/index.xhtml>

**Romania**

Sandulovici, R., Mircioiu, C., Rais, C., & Atkinson, J. (2018). Pharmacy practice and education in Romania. *Pharmacy*, *6*(1), 5. <https://doi.org/10.3390/pharmacy6010005>

*Online Pharmacies logo*. (2019). Ministerul Sănătăţii. Retrieved February 25, 2022, from <http://www.ms.ro/wp-content/uploads/2019/06/Acord-de-licenta-Logo-RO-COM-limba-engleza.pdf>

Ministerul Sănătăţii. (n.d.). *Legislatie*. Retrieved February 25, 2022, from <https://www.ms.ro/legislatie/>

Ministerul Sănătăţii. (2017). *ORDIN pentru aprobarea Normelor privind modul de calcul și procedura de aprobare a prețurilor maximale ale medicamentelor de uz uman*. Retrieved February 23, 2022, from <http://www.ms.ro/wp-content/uploads/2017/03/ORDIN-Metodologie-pre%C8%9Buri-15.03.2017-docx.pdf>

Ministerul Sănătăţii. (2008). *THE LAW OF PHARMACY*. Retrieved February 23, 2022, from <https://www.anm.ro/en/_/LEGI%20ORDONANTE/Law%20of%20Pharmacy%20no.%20266%20of%2007.11.2008.pdf>

Agenția Națională a Medicamentului și a Dispozitivelor Medicale (ANMDMR). (n.d.). *Lista medicamentelor din NOMENCLATOR*. NOMENCLATOR. Retrieved February 23, 2022, from <https://nomenclator.anm.ro/medicamente>

**Slovakia**

ŠÚKL. (2022). *Zoznam subjektov vykonávajúcich internetový výdaj*. Retrieved March 2, 2022, from <https://www.sukl.sk/hlavna-stranka/slovenska-verzia/inspekcia/lekarenstvo/internetovy-vydaj-liekov-a-zdravotnickych-pomocok/zoznam-subjektov-vykonavajucich-internetovy-vydaj?page_id=4380>

ŠÚKL. (n.d.). *Vyhľadávanie v databáze registrovaných liekov*. Retrieved March 2, 2022, from <https://www.sukl.sk/hlavna-stranka/slovenska-verzia/databazy-a-servis/vyhladavanie-liekov-zdravotnickych-pomocok-a-zmien-v-liekovej-databaze/vyhladavanie-v-databaze-registrovanych-liekov?page_id=242>

Národná rada Slovenskej republiky. (2022, January 4). *Zákon č. 362/2011 Z. z. - Zákon o liekoch a zdravotníckych pomôckach a o zmene a doplnení niektorých zákonov*. Zákony pre ľudí. Retrieved March 2, 2022, from <https://www.zakonypreludi.sk/zz/2011-362>

Zákony pre ľudí. (1950, January 1). *271/1949 Zb. - Zákon o výrobe a distribúcii liečiv*. Retrieved March 2, 2022, from <https://www.zakonypreludi.sk/zz/1949-271>

**Slovenia**

Božič, B., Obreza, A., & Atkinson, J. (2018). Pharmacy Practice and Education in Slovenia. *Pharmacy*, *7*(1), 4. <https://doi.org/10.3390/pharmacy7010004>

Agency for Medicinal Products and Medical Devices of the Republic of Slovenia (JAZMP). (n.d.). *Pricing of Medicinal Products*. Retrieved May 14, 2022, from <https://www.jazmp.si/en/human-medicines/pricing-of-medicinal-products/>

Služba Vlade Republike Slovenije za zakonodajo. (2014, March 7). *Medicinal Products Act*. Pravno-Informacijski Sistem. Retrieved May 14, 2022, from <http://www.pisrs.si/Pis.web/pregledPredpisa?id=ZAKO6295>

Služba Vlade Republike Slovenije za zakonodajo. (2008, May 9). *Rules on the classification, prescribing and dispensing of medicinal products for human use*. Pravno-Informacijski Sistem. Retrieved May 14, 2022, from [http://www.pisrs.si/Pis.web/pregledPredpisa?id=PRAV8737#](http://www.pisrs.si/Pis.web/pregledPredpisa?id=PRAV8737)

Služba Vlade Republike Slovenije za zakonodajo. (2016, December 28). *Pharmacy Practice Act*. Pravno-Informacijski Sistem. Retrieved May 14, 2022, from <http://www.pisrs.si/Pis.web/pregledPredpisa?id=ZAKO7375>

Ministrstvo za zdravje. (2022, April 13). *Mreža javne zdravstvene službe*. GOV.SI. Retrieved May 14 2022, from <https://www.gov.si/teme/mreza-javne-zdravstvene-sluzbe/>

Ministry of Health. (2022, April 4). *Retail trade in medicinal products*. SPOT.Gov.Si. Retrieved May 14, 2022, from <https://spot.gov.si/en/activities-and-professions/activities/retail-trade-in-medicinal-products/>

Služba Vlade Republike Slovenije za zakonodajo. (2009, August 10). *Rules concerning the requirements to be met by specialized shops for retail trade in medicinal products and onthe procedure for ascertaining their compliance*. Pravno-Informacijski Sistem. Retrieved May 14, 2022, from <http://www.pisrs.si/Pis.web/pregledPredpisa?id=PRAV8759>

*Centralna baza zdravil 2*. (n.d.). Centralna Baza Zdravil. Retrieved May 14, 2022, from <http://www.cbz.si/cbz/bazazdr2.nsf/Search/$searchForm?SearchView>

*Zakon o lekarniški dejavnosti*. (n.d.). Pisrs. Retrieved May 14, 2022, from http://www.pisrs.si/Pis.web/pregledPredpisa?id=ZAKO7375

**Spain**

Ministerio de Sanidad, Servicios Sociales e Igualdad. (2013). *BOE.es - BOE-A-2013-11728 Real Decreto 870/2013, de 8 de noviembre, por el que se regula la venta a distancia al público, a través de sitios web, de medicamentos de uso humano no sujetos a prescripción médica.* Agencia Estatal Boletín Oficial del Estado. Retrieved January 14, 2022, from <https://www.boe.es/buscar/doc.php?id=BOE-A-2013-11728>

Ministerio de Sanidad, Servicios Sociales e Igualdad. (2021, December 21). *BOE.es - BOE-A-2015-8343 Real Decreto Legislativo 1/2015, de 24 de julio, por el que se aprueba el texto refundido de la Ley de garantías y uso racional de los medicamentos y productos sanitarios.* Agencia Estatal Boletín Oficial del Estado. Retrieved January 14, 2022, from <https://www.boe.es/buscar/act.php?id=BOE-A-2015-8343>

Jefatura del Estado. (1997). *BOE.es - BOE-A-1997-9022 Ley 16/1997, de 25 de abril, de Regulación de Servicios de las Oficinas de Farmacia.* Agencia Estatal Boletín Oficial del Estado. Retrieved January 14, 2022, from <https://www.boe.es/buscar/doc.php?id=BOE-A-1997-9022>

Besalduch, J. (2007, July 1). *Formas de propiedad de la farmacia en España | Farmacia Profesional*. Elsevier. Retrieved January 14, 2022, from <https://www.elsevier.es/es-revista-farmacia-profesional-3-articulo-formas-propiedad-farmacia-espana-13108587>

Ministerio de Sanidad. (1997, April 25). *Ministerio de Sanidad, Consumo y Bienestar Social - Profesionales - Legislación*. Agencia Estatal Boletín Oficial del Estado. Retrieved January 14, 2022, from <https://www.sanidad.gob.es/profesionales/farmacia/legislacion/leyes/ley_16_97.htm>

AEMPS. (2020, April 15). *Información sobre la venta de medicamentos a través de sitios web y aplicaciones para móviles*. Agencia Española de Medicamentos y Productos Sanitarios. Retrieved January 14, 2022, from <https://www.aemps.gob.es/informa/campannas/medIlegales/informacion-sobre-la-venta-de-medicamentos-a-traves-de-sitios-web-y-aplicaciones-para-moviles/?lang=en>

**Sweden**

Wisell, K., & Sporrong, S. K. (2015). The Raison D’être for the community pharmacy and the community pharmacist in Sweden: a qualitative interview study. *Pharmacy*, *4*(1), 3. <https://doi.org/10.3390/pharmacy4010003>

Wisell, K., Winblad, U., & Sporrong, S. K. (2015). Reregulation of the Swedish pharmacy sector—A qualitative content analysis of the political rationale. *Health Policy*, *119*(5), 648-653. <https://doi.org/10.1016/j.healthpol.2015.03.009>

Läkemedelsverket. (2021, November 19). *Vilka regler finns gällande prissättning av receptfria läkemedel?* Retrieved February 14, 2022, from <https://fragor.lakemedelsverket.se/org/lakemedelsverket/d/vilka-regler-finns-gallande-prissattning-av-recept/>

Läkemedelsverket. (n.d.). *Retail sale of OTC medicines*. Retrieved February 14, 2022, from <https://www.lakemedelsverket.se/en/trading-pharmaceuticals/retail-sale-of-otc-medicines>

*Omreglering av apoteksmarknaden*. (2008). Riskdagen. Retrieved February 14, 2022, from <https://www.riksdagen.se/sv/dokument-lagar/dokument/statens-offentliga-utredningar/omreglering-av-apoteksmarknaden-del-1_GWB34/html>

*The Swedish Parliament*. (n.d.). Riksdagen. Retrieved February 14, 2022, from <https://www.riksdagen.se/en/>

Läkemedelsverket. (2022, January 27). *Distance trading and e‍-‍trading in OTC medicines*. Retrieved February 14, 2022, from <https://www.lakemedelsverket.se/en/trading-pharmaceuticals/retail-sale-of-otc-medicines/distance-trading-and-e-trading-in-otc-medicines>

Läkemedelsverket. (n.d.-b). *Sök läkemedelsfakta*. Retrieved February 17, 2022, from <https://www.lakemedelsverket.se/sv/sok-lakemedelsfakta?activeTab=1>

Läkemedelsverket. (2020, December 4). *Selling OTC medicines*. Retrieved February 14, 2022, from <https://www.lakemedelsverket.se/en/trading-pharmaceuticals/retail-sale-of-otc-medicines/selling-otc-medicines>

**Switzerland**

Paris, V. and E. Docteur (2007), "Pharmaceutical Pricing and Reimbursement Policies in Switzerland", *OECD Health Working Papers*, No. 27, OECD Publishing, Paris, <https://doi.org/10.1787/136157357151>

Federal Office of Public Health (FOPH). (n.d.). *Simplified supply of medicinal products subject to prescription*. Retrieved January 31, 2022, from <https://www.bag.admin.ch/bag/en/home/medizin-und-forschung/heilmittel/abgabe-von-arzneimitteln.html>

The Federal Assembly of the Swiss Confederation. (2009, January 1). *Federal Act on Medicinal Products and Medical Device*. The Federal Council. Retrieved January 31, 2022, from <https://fedlex.data.admin.ch/filestore/fedlex.data.admin.ch/eli/cc/2001/422/20190101/en/pdf-a/fedlex-data-admin-ch-eli-cc-2001-422-20190101-en-pdf-a.pdf>

Le Conseil fédéral. (2021, November 24). *Vente par correspondance de médicaments non soumis à ordonnance*. Retrieved January 31, 2022, from <https://www.newsd.admin.ch/newsd/message/attachments/69154.pdf>

Swissmedic, Swiss Agency for Therapeutic Products. (n.d.). *Selbstmedikation wird erleichtert – Anpassungen bei der Abgabe von Arzneimitteln*. Retrieved January 31, 2022, from [https://www.swissmedic.ch/swissmedic/de/home/news/mitteilungen/selbstmedikation-wird-erleichtert--anpassungen-bei-der-abgabe-vo.html#:%7E:text=Selbstmedikation%20wird%20erleichtert%20%E2%80%93%20Anpassungen%20bei%20der%20Abgabe%20von%20Arzneimitteln,-10.04.2017%20-%20Die&text=Die%20Abgabekategorie%20C%20(Apothekenpflicht)%20gibt,nicht%20verschreibungspflichtigen%20Arzneimittel%20abgeben%20k%C3%B6nnen](https://www.swissmedic.ch/swissmedic/de/home/news/mitteilungen/selbstmedikation-wird-erleichtert--anpassungen-bei-der-abgabe-vo.html#:%7E:text=Selbstmedikation%20wird%20erleichtert%20%E2%80%93%20Anpassungen%20bei%20der%20Abgabe%20von%20Arzneimitteln,-10.04.2017%20-%20Die&text=Die%20Abgabekategorie%20C%20(Apothekenpflicht)%20gibt,nicht%20verschreibungspflichtigen%20Arzneimittel%20abgebe)

Pharmapro. (n.d.). *Über den Beruf Apotheker*. Retrieved January 31, 2022, from <https://www.pharmapro.ch/de/blog/beruf-apotheker-dach.htm>

*Lists and directories*. (n.d.). Swissmedic, Swiss Agency for Therapeutic Products. Retrieved January 31, 2022, from <https://www.swissmedic.ch/swissmedic/en/home/services/listen_neu.html>

**United Kingdom**

NHS. (2021, November 18). *Dangers of buying medicines online*. Retrieved March 4, 2022, from <https://www.nhs.uk/nhs-services/prescriptions-and-pharmacies/pharmacies/dangers-of-buying-medicines-online/>

Government of UK. (2022, April 4). *Medicines: reclassify your product*. Retrieved May 17, 2022, from https://www.gov.uk/guidance/medicines-reclassify-your-product#classifications-of-medicines

Pharmaceutical Services Negotiating Committee. (n.d.). *Market Entry Regulations · Regulations · PSNC*. Retrieved March 4, 2022, from <http://archive.psnc.org.uk/pages/control_of_entry.html>

Government of UK. (2022b, April 14). *Medicines and Healthcare products Regulatory Agency*. Retrieved May 17, 2022, from <https://www.gov.uk/government/organisations/medicines-and-healthcare-products-regulatory-agency>

NHS. (2021b, November 18). *How old do you have to be to buy medicine?* NHS. Retrieved March 4, 2022, from <https://www.nhs.uk/common-health-questions/childrens-health/what-age-can-children-buy-over-the-counter-otc-medicines/>

Queen’s Printer of Acts of Parliament. (2013). *The National Health Service (Pharmaceutical and Local Pharmaceutical Services) Regulations 2013*. Legislation.Gov.Uk. Retrieved March 4, 2022, from <https://www.legislation.gov.uk/uksi/2013/349/contents>

Pharmaceutical Services Negotiating Committee. (n.d.-b). *Pharmaceutical Needs Assessment*. Retrieved March 4, 2022, from <https://psnc.org.uk/contract-it/market-entry-regulations/pharmaceutical-needs-assessment/>

Appendix 2: Establishment restrictions for pharmacy retail

| **Country** | **Distance** | **Inhabitants** |
| --- | --- | --- |
| Spain | 250 m (Regulation can be changed at the regional level) | 2,800 (It can be changed at the regional level) |
| France | N/A | Minimum 2,500 inhabitants. Afterward every 4,500 inhabitants it is possible to open a new pharmacy. the quota of 2,500 inhabitants is set at 3,500 inhabitants for the department of Guyana and the departments of Moselle, Bas-Rhin, and Haut-Rhin |
| Belgium | 1 km, 3 km, or 5 km depending on the number of inhabitants and if the existent pharmacy covers the needs of the inhabitants | The number of pharmacies per municipality should not exceed the division of the number of inhabitants by the following: a) 3,000 for municipalities with more than 30,000 inhabitants; b) 2,500 for municipalities with between 7,500 and 30,000 inhabitants; c) 2,000 for municipalities with fewer than 7,500 inhabitants |
| Luxembourg | N/A | The government according to demographic criteria issue new concessions to operate new pharmacies |
| Austria | 500 m | 5,500 inhabitants |
| Finland | When assessing the availability of medicine, the population of the area, the pharmacy services already in the area, and the location of other health care services must be taken into account | When assessing the availability of medicine, the population of the area, the pharmacy services already in the area, and the location of other health care services must be taken into account |
| Latvia | 500 m | If the population is 4,000 or less, two pharmacies may be opened, they employ a formula to estimate the maximum number of pharmacies |
| Malta | 300 m | 2,500 |
| Portugal | 350 m | 3,500 |
| Italy | 200 m | 3,300 |
| United Kingdom (England) | In the case of the UK, the control of entry restrictions are a limitation based on population needs that are decided by local health authorities | In the case of the UK, the control of entry restrictions are a limitation based on population needs that are decided by local health authorities |
| Hungary | In municipalities where there is already a public pharmacy, the establishment of a new public pharmacy may be permitted if the applicant can prove that the distance between the entrance of the existing public pharmacy and the entrance of the new public pharmacy is at least 250 meters in municipalities with a population of more than 50,000 and metropolitan districts with a population of more than 50,000, and at least 300 meters in other municipalities and other metropolitan districts | In a municipality where a public pharmacy already exists, the state health administration body may invite tenders for the establishment of a new public pharmacy if the average number of inhabitants of all public pharmacies including the new pharmacy is at least 4,000 in municipalities with more than 50,000 inhabitants and metropolitan districts with more than 50,000 inhabitants, and at least 4,500 in other municipalities and other metropolitan districts |
| Romania | N/A | in urban areas, a community pharmacy may be set up for a minimum of 3,000 inhabitants in Bucharest city and at least 3,500 inhabitants in county seat cities, and at least 4,000 inhabitants in the other cities;  in rural areas, a community pharmacy may be set up for a minimum of 4,000 inhabitants in localities over 4,000 inhabitants and no more than one pharmacy in localities under 4,000 inhabitants |
| Poland | 500 m | 3,000, if the pharmacy is located further than 1 km this restriction does not apply |
| Slovenia | The minimum distance between an existing and a new pharmacy or a branch of a pharmacy, measured on a public road, is: - in urban areas of at least 400 m, - in other areas at least 5 km | 6,000 |
| Greece | N/A | 1,000 |
| Croatia | The minimum distance between pharmacies in cities with more than 500,000 inhabitants is 200 m; the minimum distance between pharmacies in cities with 100,000-500,000 inhabitants must be 300 m, and in cities with under 100,000 inhabitants pharmacies must be 500 m apart | The demographic criterion for opening pharmacies stipulates that one pharmacy should serve 3,000 insured persons and each further pharmacy 5,000 insured persons |

Appendix 3: Summary of pharmacy chains regulation.

| **Country** | **Pharmacy chains** |
| --- | --- |
| Spain | Not allowed |
| France | Not allowed |
| Belgium | Allowed |
| Luxembourg | Not allowed |
| Germany | Not allowed, a pharmacist can own up to **3** additional branch pharmacies |
| Austria | Not allowed |
| Finland | Not allowed |
| Greece | The maximum number of licenses is limited to **10**. Pharmacy chains are not allowed |
| Estonia | Pharmacy chains outlawed in 2020, pharmacists cannot own more than **4** pharmacies |
| Lithuania | Allowed |
| Latvia | Allowed |
| Slovakia | Allowed |
| Cyprus | Not allowed |
| Malta | Not allowed to operate more than one pharmacy in the same village/town. Pharmacy chains are allowed. |
| Portugal | Ownership restriction up to **4** pharmacies. Pharmacy chains are not allowed |
| Italy | Allowed |
| United Kingdom | Allowed |
| Ireland | Allowed |
| Hungary | Ownership restriction up to **4** pharmacies. Pharmacy chains are not allowed |
| Denmark | Not allowed |
| Norway | Allowed |
| Netherlands | Allowed |
| Sweden | Allowed |
| Romania | Allowed |
| Poland | Not allowed to open new pharmacy chains, but existent can still operate. Ownership restricted to up to **4** pharmacies |
| Czech Republic | Allowed |
| Switzerland | Allowed |
| Slovenia | Not allowed |
| Croatia | Allowed |
| Bulgaria | Allowed |

Appendix 4: Examples of the application of the regulatory framework
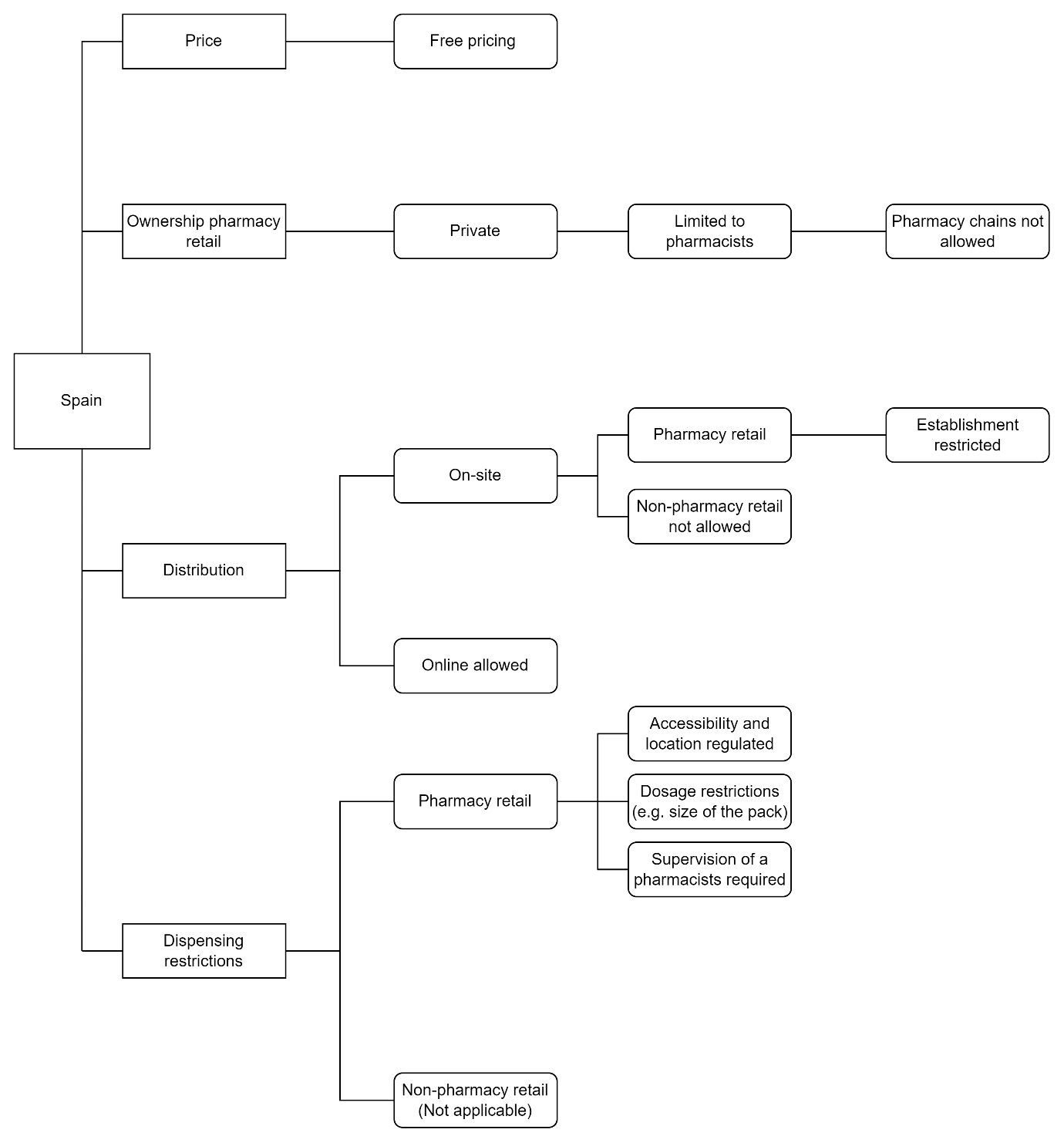

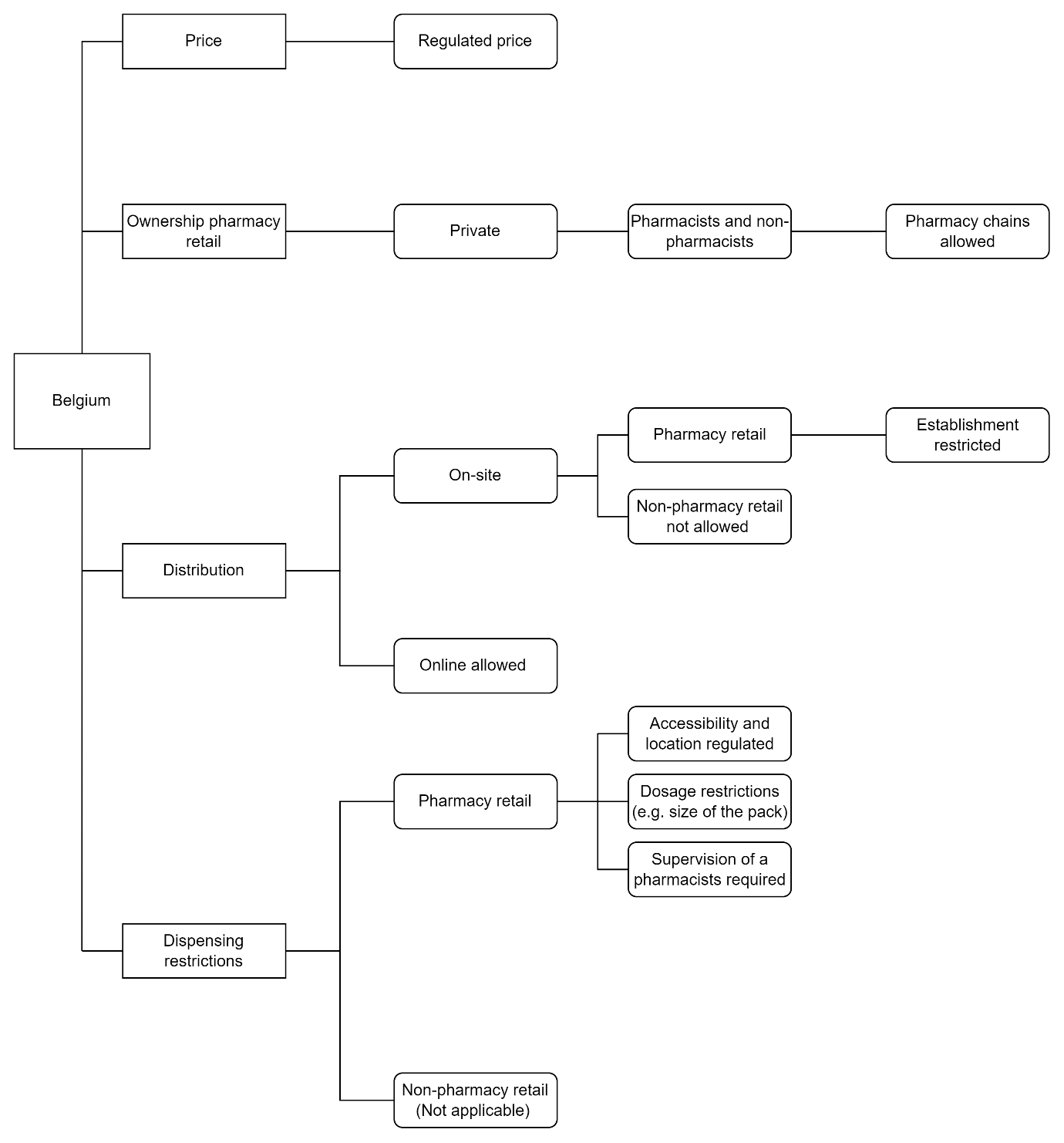

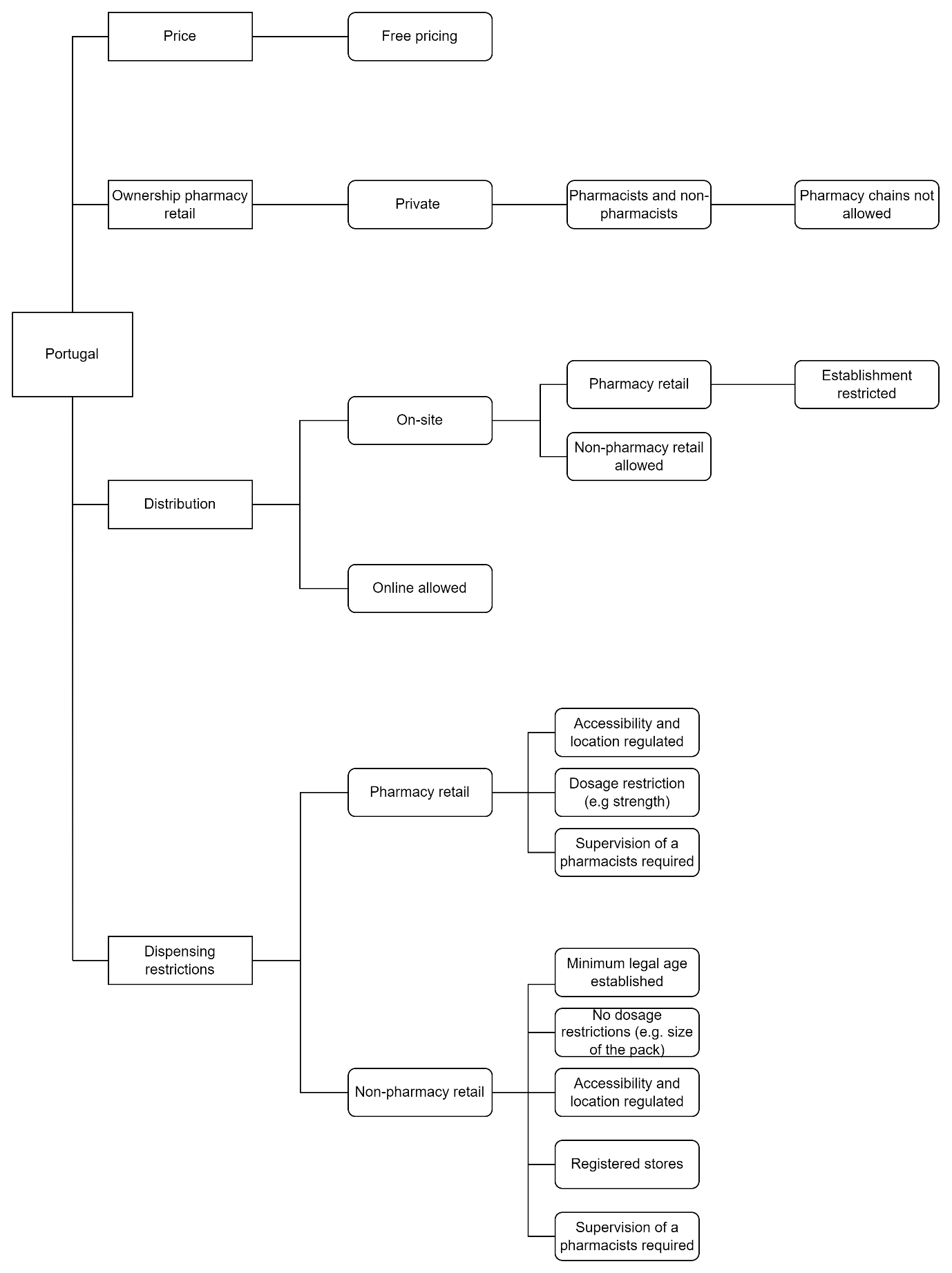


Appendix 5: Dendrograms

1. Pharmacy retail only


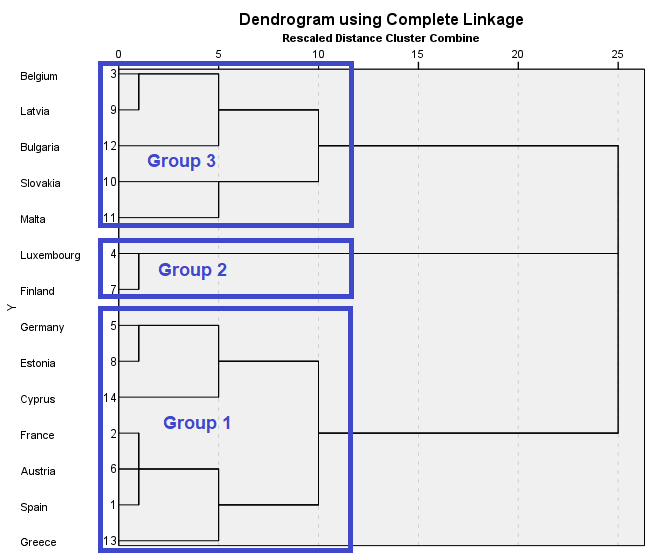


1. Pharmacy and non-pharmacy retail allowed


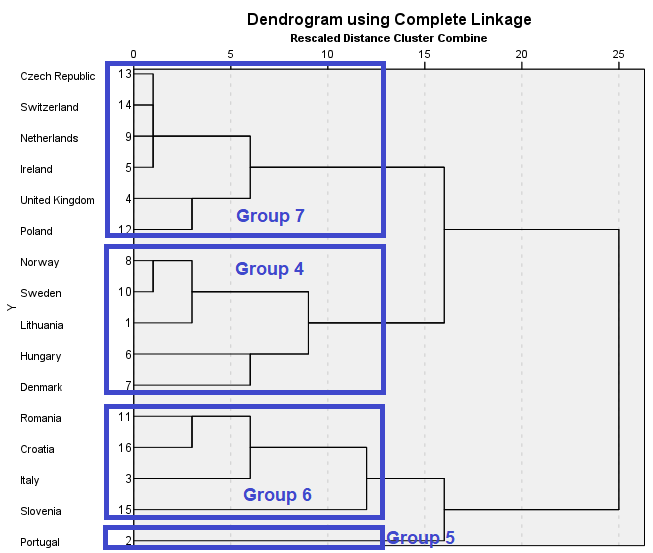

Supplement: Supplementary file 1 — Additional file 1. Appendix 1. Sources employed in the review of the regulatory framework. Appendix 2. Establishment restrictions for pharmacy retail. Appendix 3. Summary of pharmacy chains regulation. Appendix 4. Examples of the application of the regulatory framework. Appendix 5. Dendrograms. [file 40545_2023_522_MOESM1_ESM.docx]
